# First appearance deceives many: disentangling the *Hemidactylus triedrus* species complex using an integrated approach

**DOI:** 10.7717/peerj.5341

**Published:** 2018-08-02

**Authors:** Zeeshan A. Mirza, Gaurang G. Gowande, Rishikesh Patil, Mayuresh Ambekar, Harshil Patel

**Affiliations:** 1 National Centre for Biological Sciences, Tata Institute for Fundamental Research, Bangalore, Karnataka, India; 2 Department of Biotechnology, Fergusson College, Pune, Maharashtra, India; 3 Unaffiliated, Bangalore, Karnataka, India; 4 Department of Biosciences, Veer Narmad South Gujarat University, Surat, Gujarat, India

**Keywords:** *Hemidactylus subtriedrus*, *H. lankae*, Synonymy, Gekkonidae, mtDNA, Cryptic species, Taxonomy, Species complex, Mirco-CT scan

## Abstract

The gekkonid lizard genus *Hemidactylus* Oken is the second most species-rich genus of geckos with greatest diversity in the tropical regions of the world. Some species of the genus are commensal and widespread; however, there are several endemic lineages with restricted distribution. India is home to at least 35 species, with 20 endemic species and the number is steadily increasing with exploration of new habitats and integrated taxonomic approach including molecular data. We made investigations into the molecular and morphological variation throughout the distribution of *Hemidactylus triedrus* Daudin, 1802 based on fresh specimens, literature review, museum material and molecular data. Results from morphological, molecular and micro-CT based anatomical data are unequivocal and show that *H. triedrus* is a species complex represented by three species, *H. triedrus* sensu stricto and two undescribed taxa. *H. subtriedrus* Jerdon, 1854 syn. nov. was found to be morphologically similar to the type specimen of *H. triedrus*, and genetically embedded in a clade containing *H. triedrus* sensu stricto and is here treated as a junior synonym of *H. triedrus*, whereas *H. lankae* Deraniyagala is referred to as *nomen dubium* given that the types are presently not traceable and the original description is inadequate in diagnosing the taxon. The populations from western-central India and parts of Pakistan, and from southern Karnataka are distinct and diagnosable, and are herein described as two new species, respectively. Morphological and molecular data support the distinctiveness of the new species. The present work resolves a taxonomic turmoil that lasted over two centuries highlighting the need for studies that integrate morphological and molecular data.

## Introduction

Cryptic species are considered to be the worst-case scenario of taxonomic incompleteness. These are discrete species that are difficult, or sometimes impossible, to distinguish morphologically alone and are usually classified as a single species mostly spanning a wide range in its distribution (Lajmi, Giri & Karanth, 2016; Delić et al., 2017). A substantial portion of biodiversity is made up of cryptic species (Pfenninger & Schwenk, 2007) and disentangling cryptic species is imperative for assessing biodiversity and its conservation (Murthy et al., 2015). Many of these species are morphologically identical to the nominate form and an integration of multiple lines of evidence, such as molecular data, is necessary in differentiating them. Recent advances and availability of affordable, rapid DNA sequencing facilities have greatly enhanced our attempts in defining and delimiting species and numerous studies have benefitted from an integrated approach involving morphological and molecular data. Over the past five years, numerous new species of reptiles have been described from across the globe and in particular, from India and neighboring regions, using an integrated approach (Zug et al., 2006; Agarwal et al., 2016; Deepak et al., 2016; Mirza et al., 2016; Giri et al., 2017; Mirza, Bhosale & Patil, 2017).

We here present a case study of a medium-sized, conspicuously marked gekkonid lizard of the genus *Hemidactylus* Oken whose range spans from Pakistan, across the Peninsular India down to Sri Lanka (Minton, 1966; Daniel, 2002), *Hemidactylus triedrus*. The species was described based on a single specimen MNHN 2297, a male, whose origin and collector was unknown at the time of description. Subsequently the species was reported from several localities across Peninsular India, parts of Pakistan and Sri Lanka (Lesson, 1834; Boulenger, 1885, 1890; Smith, 1935; Murthy, 1990; Tikader & Sharma, 1992; Somaweera & Somaweera, 2009). Given its wide distribution and the heterogeneity of landscapes that this species occupies, it is likely that it is a species complex represented by multiple species currently grouped under *H. triedrus*.

Jerdon (1853) described an allied, questionable species or variety, *H. subtriedrus* from Nellore district, now Sri Potti Sriramulu Nellore District in Andhra Pradesh, based on the knowledge of the local Yanadees people. He considered *H. subtriedrus* and *H. triedrus* distinct (Jerdon, 1853); however, the characters presented in the description were too vague to distinguish it from the nominate form. Günther (1864) was the first to question the validity of *H. subtriedrus*, who, in his account of *H. triedrus*, states that *H. subtriedrus* is so insufficiently characterized that its distinctiveness is doubted. *H. subtriedrus* was subsequently reported from Erode district in Tamil Nadu by Stoliczka (1871, 1872) based on specimens collected by Blanford in 1871 (Blanford, 1879). Upon examination of one of these specimens, Annandale (1905) regarded it as morphologically intermediate between *H. triedrus* and *H. subtriedrus* and raised doubts on the validity of *H. subtriedrus*. The type specimen of *H. subtriedrus* was likely lost by 1905 as Annandale’s (1905) list of Indian gekkonid lizards marks *H. subtriedrus* as a species not represented in the collection of the Zoological Survey of India, then the Indian Museum. Theobald (1876) presented two accounts of the species while dealing with *Hemidactylus*, one as *H. subtriedrus* (Theobald, 1876:75) of Jerdon (1853) and later as *H. subtriedrus* (Theobald, 1876:85). This error was corrected in an erratum which stated that the account of *H. subtriedrus* on page 85 (Theobald, 1876) be merged with that of *H. subtriedrus* on page 75 (Theobald, 1876). Based on a female specimen of the then undescribed species *H. kangerensis* Mirza, Bhosale & Patil (Javed et al., 2011; Giri et al., 2017; Mirza, Bhosale & Patil, 2017) from Khammam district, Andhra Pradesh, Boulenger (1885) considered the specimen conspecific with *H. subtriedrus* and added additional characters like lamellae and labial counts which he considered to distinguish *H. subtriedrus* from *H. triedrus*. He, however, erroneously gave the type locality for the species as Ellore (now Eluru, Andhra Pradesh). Boulenger (1890) in his compilation of the herpetofauna of the region, presented the same account as in Boulenger (1885) which served as the basis of identification for subsequent revisers, notably Smith (1935), who also doubted the validity of the species. *H. subtriedrus* appeared on checklists and inventories of reptilian fauna of the Eastern Ghats of Chhattisgarh, Odisha, Telangana and northern Andhra Pradesh (Sanyal & Dasgupta, 1990; Chandra & Gajbe, 2005; Murthy & Aengals, 2008; Javed et al., 2009). This population however, is a member of the *H. maculatus* sensu lato and represents *H. sushilduttai* Giri, Bauer, Mohapatra, Srinivasulu & Agarwal or *H. kangerensis* (Agarwal, Giri & Bauer, 2011; Javed et al., 2011; Mahony, 2011; Mirza & Sanap, 2014; Giri et al., 2017; Mirza, Bhosale & Patil, 2017). Bauer et al. (2010) presented molecular data for a captive specimen of *H. subtriedrus* and two specimens of *H. triedrus* from India and Pakistan as well as a specimen from Sri Lanka and proposed validity of *H. subtriedrus*. Mahony (2011) presented a detailed discussion on the history of *H. subtriedrus* and, due to lack of conclusive morphological data and type specimens, he regarded *H. subtriedrus* as a junior subjective synonym of *H. triedrus*.

*Hemidactylus triedrus lankae* was described by Deraniyagala (1953) attributing the Sri Lankan population to this subspecies and restricting the nominate form to India. Deraniyagala (1953) distinguished “*lankae*” in bearing 13–19 precloacal femoral pores on each side separated by three non-pored scales based on a series of nine specimens vs. 6–14 in the Indian nominate form (Deraniyagala, 1953). The male holotype in the Colombo National Museum described by Deraniyagala (1953) possesses 19/18 pores, which raises doubts if it is allied to *H. triedrus*, as a review of literature and examination of museum material representing “*H. triedrus*” from Sri Lanka show that the specimens possess 7–9 pores on each side as seen in the type of *H. triedrus* (Daudin, 1802; Somaweera & Somaweera, 2009; Batuwita & Pethiyagoda, 2012). It is likely that the type series of *H. lankae* comprised of multiple species that did not include representatives of *H. triedrus* as the specimens possess femoral pores >13. De Silva (1957) collected series of specimens from Sri Lanka which he attributed to *H. triedrus triedrus*. Among these specimens, he attributed one to the nominate form from India as it possessed 7/7 pores separated by a single non-pored scale and mentioned that the second population bore 12–19 pores separated by three non-pored scales. Bauer et al. (2010), in addition to proposing the validity of *H. subtriedrus* without any morphological data raised the *H. triedrus lankae* to a species rank. Nonetheless, the types of *H. triedrus lankae* are missing or lost (Amarasinghe et al., 2009) and hence identification of current material assigned to *H. lankae* is not possible. The nomenclature status of *H. lankae* is currently unstable and the absence of a type specimen complicates matters and is here referred to as *nomen dubium*, pending a detailed revision based on material from across Sri Lanka (see Discussion).

In order to verify the validity of *H. subtriedrus* proposed by Bauer et al. (2010) and to put an end to the taxonomic confusions surrounding *H. subtriedrus*, we collected a male specimen from Nellore district and compared it with the existing specimens in the collection of the museums, which also includes the type of *H. triedrus*. Additionally, specimens of *H. triedrus* were collected near Pondicherry and the outskirts of Chennai which might have been the localities from where the type of *H. triedrus* originated and a few from Maharashtra, Karnataka, Andhra Pradesh and Gujarat. The specimen from Nellore is identical to the specimens collected from Pondicherry and Chennai and match the type of *H. triedrus* confirming that *H. subtriedrus* is a synonym of *H. triedrus*. The synonymy allows us to restrict the type locality of *H. triedrus* to Nellore and provide a detailed description of the species based on fresh as well as museum material. Restriction of type locality for *H. triedrus* is here deemed necessary as this group is a species complex and to avoid further taxonomic confusion. The specimen from Nellore agrees in all respect with the available description and the type of the species in accord with the International Code for Zoological Nomenclature (International Commission on Zoological Nomenclature (ICZN), 2014). Additionally, the specimens from western-central India and Pakistan are distinct from *H. triedrus* and so are specimens from Karnataka which are herein described as two new species based on morphological as well as molecular data.

## Materials and Methods

### Morphological and meristic data

Specimens were collected and manipulated with the authorization and under strict control and permission of the governments of India (Ministry of Environment, Forest and Climate Affairs, MoEF) and the forest department of concerned states. Specimens were captured and processed following the guidelines and protocols stated in the collecting permits (B/WPS/8/9388-92/2013-14, 71/16/4454 and 71/16/4487) and agreements obtained from the competent authorities. All efforts were made to minimize animal suffering. Specimens were captured by hand in field and euthanized with Halothane following standard animal euthanasia guidelines (Leary et al., 2013), and fixed in 6% formaldehyde buffer. The specimens were later washed and stored in 70% ethanol, and deposited in the collections of the Collection facility of the National Centre for Biological Sciences, Bangalore and the Bombay Natural History Society, Mumbai. Comparative material was directly examined from accessible museums or high resolution images of specimens were acquired to confirm identification. Specimens were collected from unprotected areas like private farm houses and agricultural field and not from protected areas. Specimen collection was kept to minimal as a large part of the study was based on existing material in natural history museums.

All measurements were taken following Mirza & Sanap (2014) with Mitutoyo™ digital calipers (Mitutoyo Corporation, Kawasaki, Japan) (to the nearest 0.1 mm): snout-vent length (SVL; from tip of snout to vent), trunk length (TRL; distance from axilla to groin measured from posterior edge of forelimb insertion to anterior edge of hind limb insertion), body width (BW; maximum width of body), crus length (CL; from base of heel to knee); tail length (TL; from vent to tip of tail), tail width (measured at widest point of tail); head length (HL; distance between retroarticular process of jaw and snout-tip), head width (HW; maximum width of head), head height (HH; maximum height of head, from occiput to underside of jaws), forearm length (FL; from base of palm to elbow); ear length (EL; longest dimension of ear); orbital diameter (OD; greatest diameter of orbit), nares to eye distance (NE; distance between anteriormost point of eye and nostril), snout to eye distance (SE; distance between anteriormost point of eye and tip of snout), eye to ear distance (EE; distance from anterior edge of ear opening to posterior corner of eye), internarial distance (IN; distance between nares), interorbital distance (IO; shortest distance between left and right supraciliary scale rows) (Table 1). Meristic counts and external observations of morphology were made using a Leica™ S8APO (Leica Camera, Wetzlar, Germany) dissecting microscope. Images of the specimens were taken with a Canon™ 70D mounted with a Canon™ 100 mm macro illuminated with two external Canon™ 430EX-II flashes (Canon Inc., Tokyo, Japan). Zoobank LSID for the manuscript is urn:lsid:zoobank.org:pub:28E6F879-6559-4CE3-9BE9-5A51F10EAD26.

**Table 1:** Morphological and meristic data for specimens of *Hemidactylus whitakeri* sp. nov.

Specimens number	NCBS AU712	NCBS AU713	NCBS AU719	NCBS AU720	BNHS 1827	BNHS HTK2/2015	BNHS HTK1/2010
	Holotype	Paratype	Paratype	Paratype	–	–	–
Sex	♀	♀	♂	♂	♀	♂	♀
SVL	53.7	45	68.2	57.3	53.2	58.1	59.9
TRL	26.8	18.9	30.1	25.5	24.2	23.8	26.6
BW	12	10	16.3	12.5	14.4	13.2	14.8
CL	7.7	6.4	10.5	9.2	8.9	8.9	9.1
TL	65	5.4*	66.8	27.9*	30.65*	23.2*	20.11*
TW	6.6	36*	8.2	7.1	6.4	6.5	8.1
HL	12.2	9.9	14.8	13.2	17.0	18.2	17.8
HW	10.7	9.8	15.2	12.2	11.4	13.0	12.6
HH	7.4	6.1	9.2	8.1	5.9	7.1	7.2
FL	7.8	5.1	8.6	8.3	7.9	8.6	8.9
OD	3.1	2.4	3.9	3.0	3.8	3.6	4.3
NE	4.6	3.3	5.7	4.7	5.1	4.8	5.6
SE	6.5	5.4	7.7	6.4	6.6	7.2	7.8
EE	5.1	3.5	6.0	5.3	4.8	5.0	5.0
EL	0.8	0.6	1.2	0.5	1.1	1.8	1.8
IN	1.3	1.7	2.1	2.6	1.7	1.9	1.9
IO	4.6	3.7	5.6	4.8	6.1	6.4	6.7
Pores L	–	–	7	8	–	8	–
Pores R	–	–	7	7	–	7	–
gap btw pores	–	–	3	3	–	3	–
**Lamellae**							
L manus	7-9-9-9-9	7-8-8-8-8	7-9-10-9-9	6-8-8-9-8	–	–	–
R manus	7-9-9-9-9	7-8-8-8-8	7-9-9-9-9	6-8-8-9-8	–	–	–
L pes	7-10-10-9-8	7-9-9-9-8	8-9-10-9-9	6-9-9-9-8	–	–	–
R pes	7-10-10-9-8	7-9-9-9-8	8-9-10-8-9	6-9-10-9-9	–	–	–
Supralabials L/R	8/9	8/8	9/8	8/9	–	–	–
Infralabials L/R	7/7/	7/7	8/8	8/8	–	–	–

**Note:**

* Indicates broken or regenerated tail.

Micro-CT scans were generated for three male specimens using a Bruker^®^ Skyscan 1272 (Bruker BioSpin Corporation, Billerica, MA, USA). Head of the specimens were scanned from 16 to 20 min at 15 μm. Volume rendering was performed with CTVox (Bruker BioSpin Corporation, Billerica, MA, USA) and images were edited in Adobe Photoshop CS6. Osteological description is based on volume renders retrieved from CTVox following terminology of the skull described by Evans (2008).

Institutional acronyms used in the manuscript are as follows: BNHS—Bombay Natural History Society, Mumbai; CAS—Californian Academy of Sciences, San Francisco; CES—Centre for Ecological Sciences, Bangalore; CM—Herpetology collection, Carnegie Museum of Natural History, Pittsburgh; MNHN—Muséum national d’Histoire naturelle, Paris; NHM—Natural History Museum, London; NCBS—Collection facility, National Centre for Biological Sciences, Bangalore; USNM—National Museum of Natural History, Washington; WHT—Wildlife Heritage Trust, Colombo; ZSI—Zoological Survey of India, Kolkata. Comparative material examined is listed in Supplemental Material S3.

### Molecular analysis

Genomic DNA was extracted from liver or tail tissue using HiMedia™ mammalian genomic DNA extraction kit following protocol directed by the manufacturers. We amplified partial segments of two mitochondrial genes, Nicotinamide adenine dinucleotide dehydrogenase: ubiquinone oxidoreductase core subunit 2 (*ND2*) using newly designed primers- Liz_ND2F 5′- ACCTGACTTAGCCTAGAAC-3′, Liz_ND2R 5′- TAGGTTGAGTTGTATAGTGC-3′ and cytochrome b (*cyt b*) with published primers *Cytb1* (5′-CCATCCAACATCTCAGCATGATGAAA-3′) and *Cytb2* (5′-ACTGTAGCCCCTCAGAATGATATTTGTCCTCA-3′) for *cyt b* (Kocher, Thomas & Meyer, 1989). A 25 μl reaction was set containing 11.5 μl of Qiagen™ TopTaq PCR Master Mix, 11.5 μl of water, 0.5 μl of each primer and one μl template DNA, carried out with an Eppendorf Mastercycler Nexus GSX1. Thermo-cycle profile used for amplification were as follows: 94 C for 5 min, (denaturation temperature 94 °C for 30 s, annealing temperature 45 °C for 50 s, elongation temperature 72 °C for 1 min) × 35 cycles, 72 °C for 10 min, hold at 4 °C for *ND2* and for *cyt b* as described by Mirza, Bhosale & Patil (2017). PCR product was cleaned using Qiagen™ PCR Purification Kit and sequenced with a 3730 DNA Analyzer. Sequences for *ND2*, *cyt b* and two nuclear genes Recombination activating gene 1 (*RAG1*) and Phosducin (*PDC*) gene of selected *Hemidactylus* spp. of the “*brookii* group” of Bauer et al. (2010) were downloaded from GenBank and selection of taxa follows Mirza, Bhosale & Patil (2017) (Table S1). Downloaded sequences were aligned in MEGA6 (Kumar, Stecher & Tamura, 2016) using ClustalW (Thompson & Gibson, 2003) with default settings. For optimal partitioning strategy and evolutionary substitution model, aligned data was analyzed using PartitionFinder v.1.1.1 (Lanfear et al., 2012). Maximum Likelihood (ML) method was implemented to assess phylogenetic relationship with RAxML GUI (Silvestro & Michalak, 2012). Data were partitioned and sequence substitution model was based on the optimal partitioning scheme suggested by PartitionFinder (Table S2). ML analysis was run for 1,000 non-parametric bootstrap replicates with rapid ML search option. Bayesian Inference (BI) was implemented in MyBayes 3.2.2. (Ronquist & Huelsenbeck, 2003) and was run for 10 million generations. The analysis reached a standard split frequency of <0.005 and was not continued further. A total of 25% percent of trees generated were discarded as burn-in. Effective sampling size was checked in Tracer v. 1.6 to ensure that values for each parameter exceeded 200 (Rambaut et al., 2014) for BI. Sequence divergence uncorrected “*p*-distance” was calculated in MEGA6 for *cyt b* and *ND2* (Tables S6 and S7). For the ML + BI analyses, *H. flaviviridis* Rüppell was chosen as the outgroup taxon to root the phylogenetic trees. Individual gene trees were used to infer phylogenetic relationships as tree topologies were identical for *ND2, RAGI* and *PDC*. Data for these three genes were concatenated to assess phylogenetic relationships, whereas a single gene tree for *cyt b* data is presented in the Supplemental Material.

Genealogical relationships within the *H. triedrus* species complex were analyzed using a haplotype network to further attest our findings from molecular phylogenetics. For the sake of constructing haplotype networks, we treated the three species as three populations of *H. triedrus*. A haplotype data file of *cyt b* data of only the members of the *H. triedrus* complex was generated using DnaSP v6 (Rozas et al., 2017). Sites with gaps/indels or missing data were not considered, invariable sites were removed, and measures such as the number of haplotypes, haplotype diversity and the total number of variable sites were calculated. The resultant haplotype file contained eight haplotypes of 48 bp length each. This haplotype data file was used in the construction of haplotype network in PopART (http://popart.otago.ac.nz) (Leigh & Bryant, 2015) using the Templeton, Crandall and Sing (TCS) method (Clement et al., 2000).

### Species delimitation

Bayesian Poisson Tree Process (bPTP) based on evolutionary placement algorithm was implemented on the web server (http://species.h-its.org/ptp/) following Zhang et al. (2013) for inferring putative species. ML tree based on *ND2* gene was supplied for the analysis. The outgroup, *H. flaviviridis*, was excluded from the analysis for optimum results. The analysis was run for 100,000 generations with three chains and 25% of the trees were discarded as burn-in. Results of the analysis are presented in Fig. S1 and Table S5.

### Nomenclature acts

The electronic version of this article in portable document format will represent a published work according to the International Commission on Zoological Nomenclature (ICZN), and hence the new names contained in the electronic version are effectively published under that Code from the electronic edition alone. This published work and the nomenclatural acts it contains have been registered in ZooBank, the online registration system for the ICZN. The ZooBank LSIDs (Life Science Identifiers) can be resolved and the associated information viewed through any standard web browser by appending the LSID to the prefix http://zoobank.org/. The LSID for this publication is: urn:lsid:zoobank.org:pub:28E6F879-6559-4CE3-9BE9-5A51F10EAD26. The online version of this work is archived and available from the following digital repositories: PeerJ, PubMed Central and CLOCKSS.

## Results

**Figure 1:**
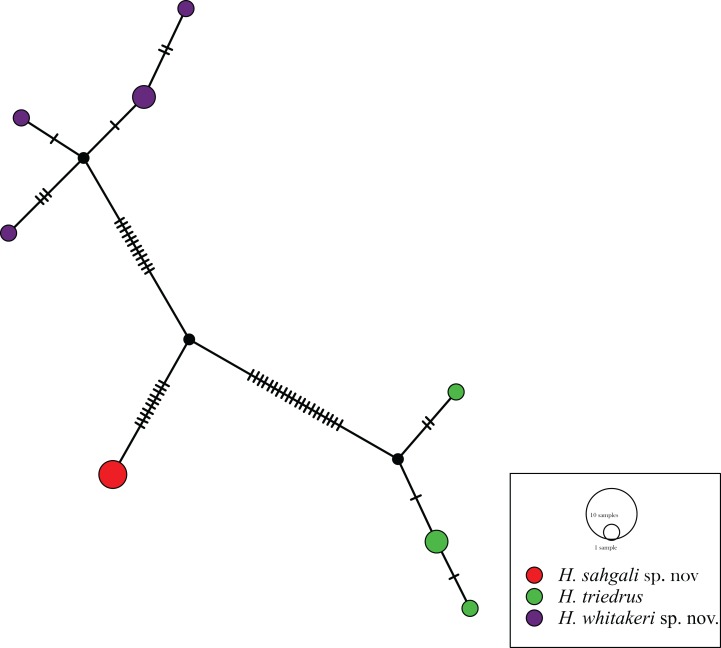
Haplotype network based on mitochondrial *cyt b* gene for members of *H. triedrus* species complex.

The 12 sequences of *H. triedrus* fell into eight haplotypes, with a haplotype diversity of 0.9242. A sequence from Pakistan, one from an unknown locality from India, and another sequence from the Deccan Traps region was recovered as a single haplotype, Hap_1, which here represents *H. sahgali* sp. nov. The Sri Lankan population was recovered as a unique haplotype (Hap_2), which was closely related to the central-eastern and eastern coastal population (Hap_3, Hap_4). This cluster of haplotypes includes a sequence from Nellore, and represents *H. triedrus* sensu stricto, by the virtue of restriction of type locality. The Bangalore population fell into four closely related haplotypes, this population is herein treated as *H. whitakeri* sp. nov. Fig. 1). Very shallow divergence was observed within the clusters, whereas all the clusters were greatly separated from each other. Haplotype network further lends support to our recognition of the three populations as distinct species. Morphological and skull anatomical data also supports distinctiveness of the three lineages which are here treated as three species, of which, two are described in this communication.

**Figure 2:**
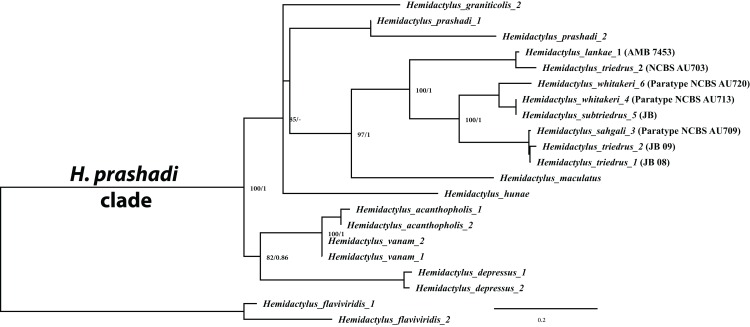
Molecular phylogeny based on concatenated mitochondrial *ND2* and two nuclear (*RAG1 + PDC*) genes for members of the *H. prashadi* group of Bauer et al. (2010) rooted with *H. flaviviridis* as the outgroup. Sequence names correspond to their original publications which generated them.

Molecular phylogenetics based on concatenated data of 2,405 bp consisting of one mitochondrial *ND2* gene (966 bp) and two nuclear genes, *RAG1* (1,044 bp) and *PDC* (395 bp) recovered three divergent, well supported clades (bootstrap support 100% + BI support 1), supporting the hypothesis that *H. triedrus* represents a species complex (Fig. 2), thus refuting a single species taxonomy. Clade one represents *H. triedrus* sensu stricto that includes sequences from Nellore, Pondicherry and Sri Lanka, clade two contains sequences form Karnataka which is here described as *H. whitakeri* sp. nov. and clade three contains sequences from Maharashtra, Gujarat and parts of Pakistan described as *H. sahgali* sp. nov. Although the topology recovered from *cyt b* (Fig. S3) and concatenated dataset are different, the three clades are always recovered as distinct, with good to high support. Additional support to the claim is the presence of nine indels in *cyt b* sequences of *H. sahgali* hinting that it represents a distinct evolutionary lineage (Fig. S2). Results from bPTP analysis further support the distinctness on the three species (Fig. S1; Table S5).

## Systematics

### *Hemidactylus triedrus* species group

*Species included: H. triedrus*, *H. sahgali* sp. nov., *H. whitakeri* sp. nov.

*Definition:* Members of the group grow to a medium size ranging from 45–74 mm in SVL with a rather robust habitus. Dorsum with distinct dark bands may be edged with white or lighter shade of brown. Scales on the dorsal aspect of trunk granular intermixed with large keeled sub-trihedral or trihedral tubercles arranged in 15–20 fairly regular rows. Supralabial eight to nine and infralabials seven to eight to angle of jaw. Lamellae on digit one of manus and pes range from seven to eight and on digit four of manus and pes range from 8–10. Males possess a series of 7–15 precloacal femoral pores interrupted medially by a diastema of one to three non-pored scales. A single or a pair of sub-conical to rounded post cloacal spur. Tail with usually eight keeled tubercles in a whorl on segment I, the number subsequently reduces with progression of tail segments.

*Distribution:* Widespread across dry zones of India and Sri Lanka and parts of Pakistan.

**Figure 3:**
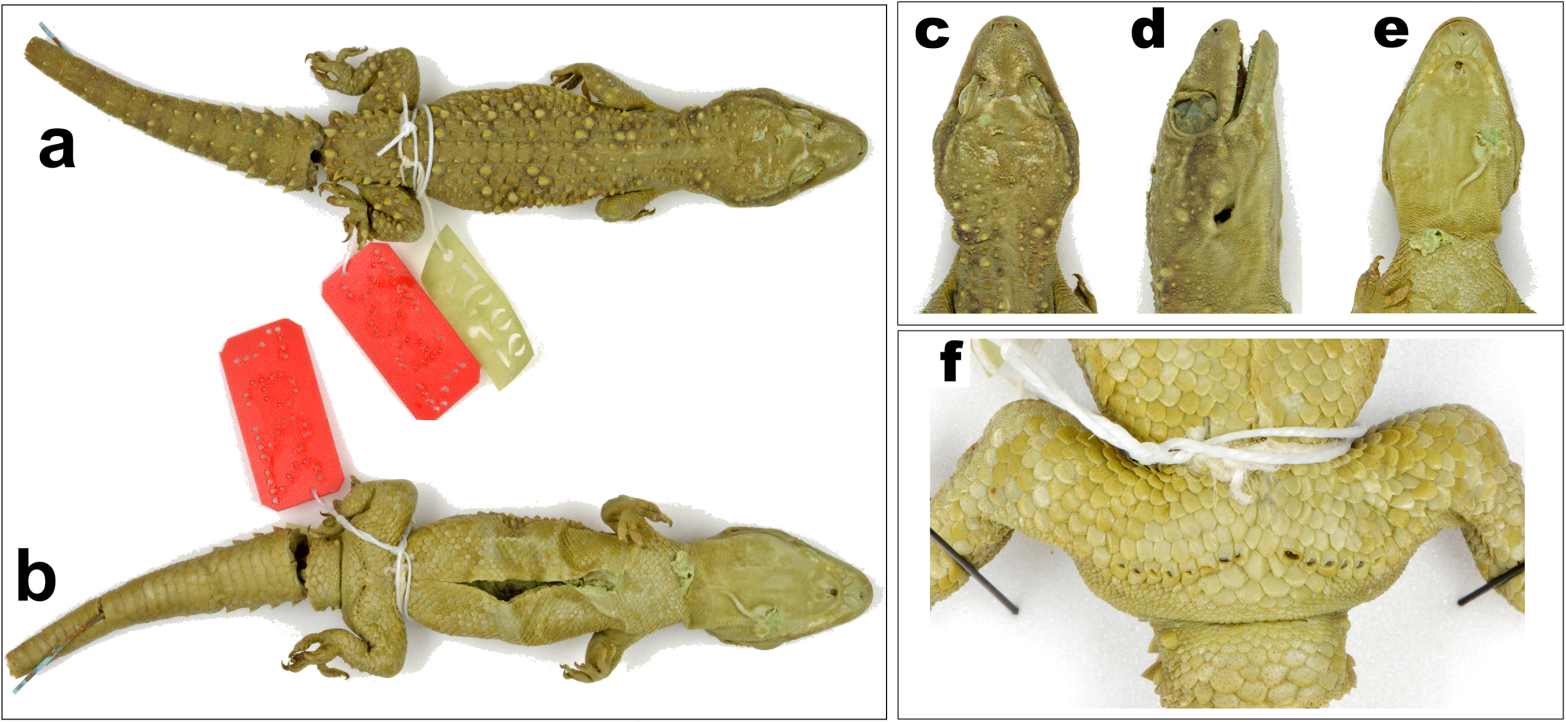
Male holotype of *H. triedrus* MNHN 2297. (A) dorsal aspect, (B) ventral aspect, (C) dorsal aspect of head, (D) lateral aspect of head, (E) ventral aspect of head, (F) cloacal region showing pre-cloacal femoral pores.

**Figure 4:**
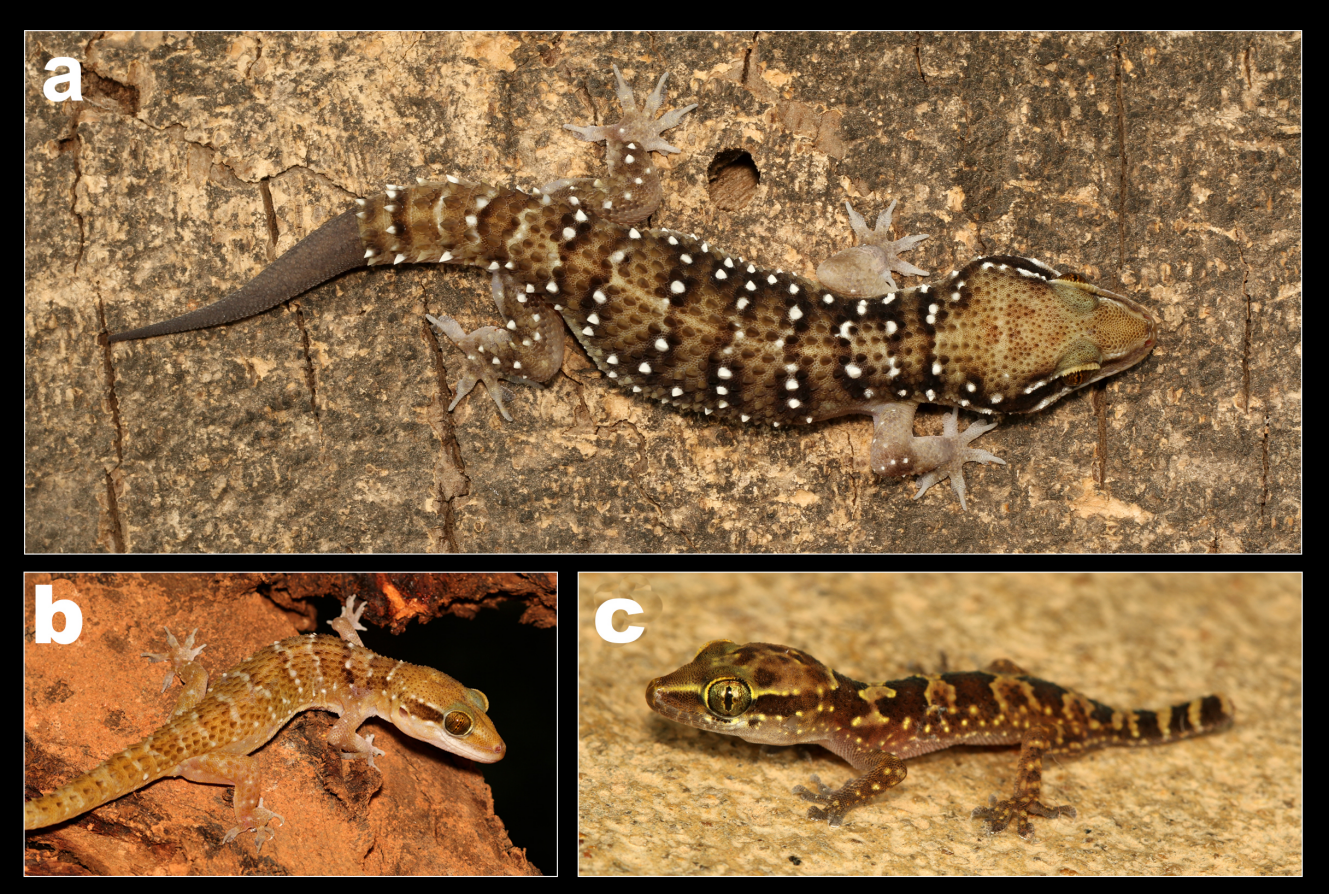
Coloration in life of *H. triedrus*. (A) male NCBS AU703 from Nellore, (B) an uncollected female from Pondicherry, (C) uncollected juvenile from Pondicherry.

**Figure 5:**
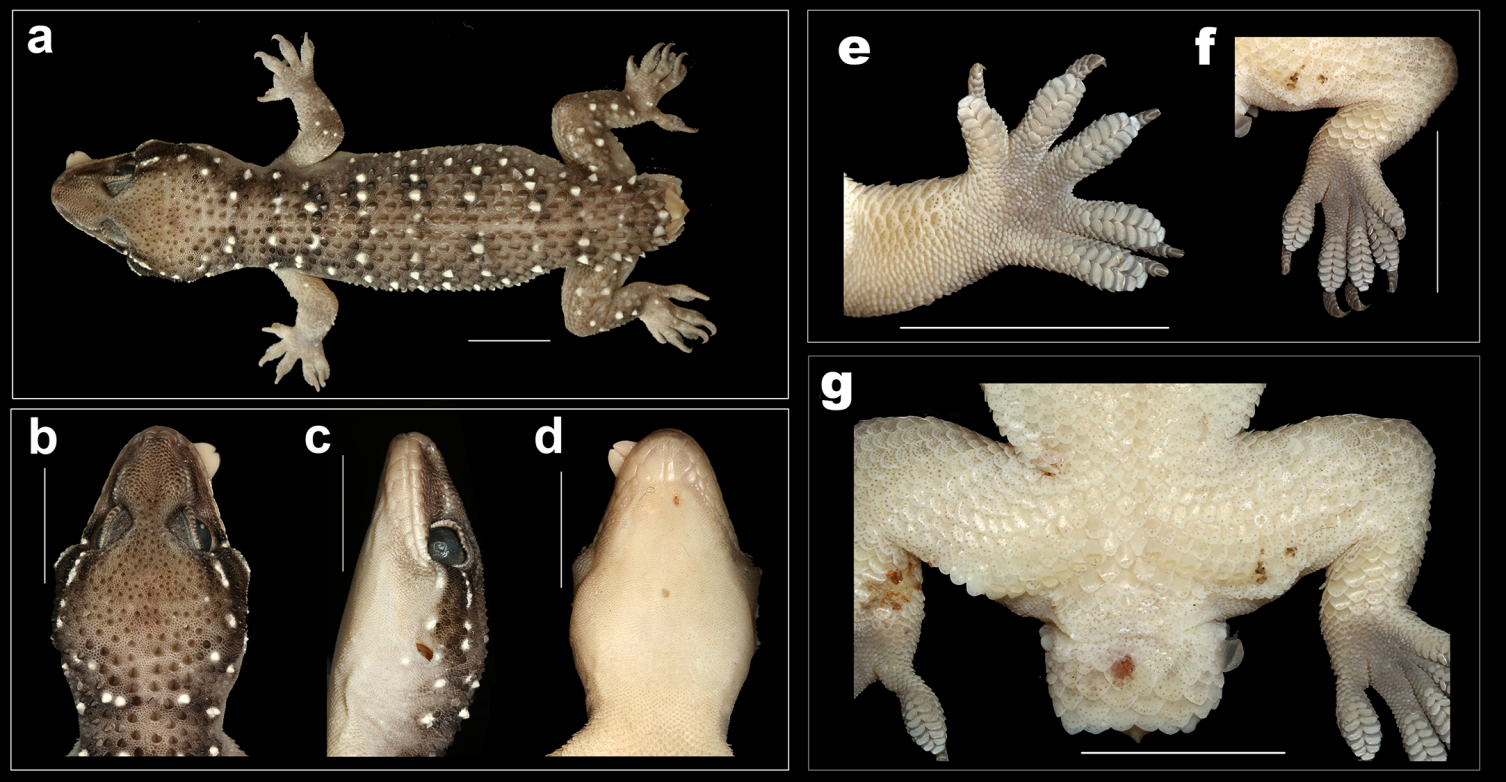
Male *H. triedrus* NCBS AU703 from Nellore. (A) dorsal aspect, (B) dorsal aspect of head, (C) lateral aspect of head, (D) ventral aspect of head, (E) left manus, (F) left pes, (G) cloacal region showing pre-cloacal femoral pores. Scale bar 10 mm.

***Hemidactylus triedrus*Daudin, 1802**
*Gecko triedrus* Daudin, 1802:155
*Hemidactylus triedrus* Lesson, 1834:311; Boulenger, 1885:133 (in part); Smith, 1935:88 (in part)
*Hemidactylus subtriedrus* Jerdon, 1854:467; Smith, 1935:89 syn. nov.
*Hemidactylus triedrus lankae* Somaweera & Somaweera, 2009:180
*Hemidactylus lankae* Bauer et al., 2010:350
Figs. 3–5; Table S3.

*Holotype*: male, MNHN 2297, from an unknown locality and collector (Fig. 3).

*Type locality (here restricted)*: Nellore, Sri Potti Sriramulu Nellore District, Andhra Pradesh following the synonymy of *H. subtriedrus*.

*Material examined*: INDIA – female CAS 104151, Nagercoil, Kanyakumari district, Tamil Nadu; male BNHS 1668 Kalakkad Mundanthurai Tiger Reserve, Tamil Nadu; male BNHS 849 Kanyakumari district, Tamil Nadu; three females BNHS 683, BNHS 683-1, BNHS 1110 Palghat, Kerala; male NCBS AU703, Donthali near Nellore, Sri Potti Sriramulu Nellore District, Andhra Pradesh; male NCBS AU705, (near Pondicherry) Aranya forest and sanctuary, Poothurai Rd, Thiruchitrambalam, Tamil Nadu (11.962908°N, 79.767136°E); two males NCBS AU706–707, Chengalpettu, Tamil Nadu; male NCBS AU704 near Sri Varahalakshmi Narasimha Swamy Vari Devasthanam, Visakhapatnam, Andhra Pradesh (17.762971°N, 83.256334°E). Sri Lanka: male WHT 2161, Virankattuwa; males CM 67601–67603, Poḷonnaruwa, North Central Province; undetermined sex CM 67801 Tabbowa, Puttalam district, North Western province; male CM 83615 Marichchukkaddi, Northern province; male USNM 120313, Matale District, Central Province; male USNM 254547 & three females USNM 254549–USNM 254551, Trincomalee, Eastern Province; male USNM 254583, Anuradhapura District, North Central District; male USNM 267726, Mannar, Northern Province; females USNM 120311, Rattota, Clodagh Estate, Kandy District, Central Province; three females USNM 254664–USNM 254666 Mundel, three mi from, Nawadamkulama, Puttalam, North Western province; four females USNM 254703, USNM 254727—USNM 254729 Marichchukkaddi, Mannar, Northern Province; female USNM 267725 Mahiyangana, Badulla District, Uva; female USNM 267727 Parayannalankulam Rest House, Mannar, Northern Province.

*Diagnosis*: A medium sized fairly stout gecko, adults ranging 58–76 mm in SVL. Dorsum in a shade of light brown with paired, thin black edged white bands at regular intervals. Dorsal scalation on trunk, granular, intermixed with enlarged, keeled 19–20 trihedral tubercle rows arranged in fairly regular longitudinal series. Seven lamellae under digit I of pes and manus, eight to nine under digit four of manus and pes. An angular series of seven to nine precloacal femoral pores separated at mid-pelvic by a diastema of one to three non-pored scales.

*Genetic divergence*: Genetic divergence within *H. triedrus* is 1–2% for *cyt b*, whereas divergence from *H. sahgali* sp. nov. and *H. whitakeri* sp. nov. is 14% and 14–16% respectively. Divergence from *H. sahgali* sp. nov. and *H. whitakeri* sp. nov. for the gene *ND*2 is 17–18% each. The two sequences of *H. triedrus* are 3% divergent from each other for *ND2*.

*Description of male NCBS AU703 from Nellore* (Figs. 4A and 5): The specimen is in good condition, preserved in a linear manner lacking the entire tail, which broke post preservation (Fig. 5A).

A medium sized gecko (SVL 72 mm) with a fairly large head (HL/SVL ratio 0.28), head slightly longer than wide (HW/HL ratio 0.80), head depressed (HH/HL ratio 0.54), distinct from neck (Figs. 5B–5D); canthus rostralis slightly inflated; snout short (SE/HW ratio 0.53), obtusely pointed from dorsal view and acutely in lateral view (Figs. 5B and 5C); longer than eye diameter (OD/SE ratio 0.48); scales on the snout subequal, convex, those anterior to the eye and on canthus rostralis, larger than the surrounding scales; eyes large (OD/HL ratio 0.20), pupil vertical with crenulated edges; supraciliaries larger on the anterior edge of the orbit, gradually decreasing in size as they progress toward the posterior portion of the orbit; ear-opening large, sub-oval, obliquely oriented, length less than half of the orbital diameter (EL/OD ratio 0.37), lobules absent; eye to ear distance greater than diameter of eye (EE/OD ratio 1.70); rostral quadrangle, much wider than deep, divided by a median suture for its entire length; rostral in contact with nasal, first supralabial and internasals; two large and a slightly smaller internasal between nasals; mental triangular, slightly wider (3.72) than long (2.98); two pairs of postmentals, anterior postmental longer (2.7) than wide (1.7); posterior pair of postmental smaller than anterior pair, wider (1.3) than long (1.6); anterior postmental in contact with mental, infralabials one & two, and posterior pair of postmental; posterior postmentals less than half the size of the anterior one; anterior postmental equal to the width to the first infralabial; posterior postmental half the width of second infralabials; posterior postmental in contact with anterior postmental, first as well as second infralabial; posterior borders of postmentals aligned in a straight line (Fig. 5D); scales on throat and those posterior to the postmentals circular, smaller than the ones ventral aspect of trunk; supralabials (to midorbital position) ten on left and right side; supralabials (to angle of jaw) 12 on left as well as right side; infralabials (to angle of jaw) nine on either sides.

Body elongate (TRL/SVL ratio 0.42) and dorsoventrally flattened; lacking distinct ventrolateral furrow; dorsal scalation on trunk granular intermixed with enlarged, keeled, trihedral tubercles, fairly arranged in 19–20 longitudinal rows; dorsal tubercles on mid-dorsum situated in close proximity, slightly depressed, slightly longer (1.4) than wide (1.3); tubercles on the lateral aspect of the trunk larger, slightly spaced out in comparison with tubercles on mid-dorsum; ventral scales on trunk smooth, flat, larger than dorsal scales; mid body scales across belly 30–32; seven (left) and seven (right) femoral pores separated at mid-pelvic region by a diastema of three non-pored scales; size of non-pored scales equal to pored scales (Fig. 5G).

Limbs moderately long, stout; digits dilated, bearing horizontally oriented lamellae on ventral surface; lamellae on basal half of digit I of manus and pes undivided, lamellae on rest other digits divided (excluding terminal lamellae); clawed, claw slightly smaller than length of the lamellar region; forelimbs short (FL/SVL ratio 0.14), slightly shorter than hind limbs (CL/SVL ratio 0.15). Terminal phalanx of all digits curved, arising angularly from distal portion of expanded lamellar pad, free portion of phalanx of all digits half to more than half long as the dilated portion. Lamellae beneath the digits, 7-8-8-8-8 (Fig. 5E) on left as well as right manus; left pes 7-9-9-9-8, right pes 7-9-9-8-8 (Fig. 5F). Lamellae not reaching the base of the digit IV of pes, covering 80% of the digit. Relative lengths of digits: III>V>IV>II>I (left manus), V>II>IV>III>I (left pes).

Tail moderately depressed, oval in cross section, longer than SVL (TL/SVL ratio 1.21), 42.5 mm of the tail regenerated. Caudal segments distinct; pholidosis of original tail dorsum with small, juxtaposed scales intermixed with large keeled trihedral tubercles, scales on regenerated portion of tail homogenous, slightly smaller compared to the scales on original tail and lacking tubercles. First tail segment with a whorl of eight large conical, keeled tubercles, second segment onward, each segment with six tubercles. Ventral aspect with large, broad scales covering about ∼60% of the tail width from base of tail to the tip. A single sub-conical post cloacal spur.

*Coloration in preservative:* Dorsum in a shade of light brown with black edged white bands at regular intervals. The first band is present on the nape and is not complete, interrupted medially at the vertebral region, second pair of the band is present on the intersection of the forelimbs, two pairs on the trunk and the last pair on the intersection of the hindlimbs. The white coloration of the bands is present on the tubercles which is surrounded by black colored scales. A white stripe running from the nares along the canthus rostralis to the ear opening, interrupted by adjoining dark brown coloration posterior to the eye. Limbs much paler nearly whitish pink in color with a few white tubercles. Ventrally off-white with each scale bearing sparse black speckles.

*Coloration in life:* Background coloration on the head, trunk and tail light brown with paired bands from the nape all through the trunk to the tip of the tail. The bands are formed of a central white band edged with blackish brown on its anterior and posterior portion. The white central portion in these bands in mostly restricted to the tubercles which are bright white. Limbs pinkish in shade with a few white colored tubercles. Hind limbs slightly in a shade of brown and are darker compared to the anterior limbs. A yellowish stripe runs from mid canthus rostralis through the supraciliary scales up till the supra-tympanic region. Ventrally white.

*Variation observed in examined specimens:* All examined specimens match the topotype from Nellore except for morphometric data presented in Table S3. Specimens range in SVL from 58–76.6 mm. Pre-cloacal femoral pores range from seven to nine separated medially by one to three non-pored scales in males. Coloration in life is also quite variable, some individuals are dark with bright white tubercles (Fig. 4A) whereas some individuals are paler in coloration and lack distinct white colored tubercles (Fig. 4B). Juveniles are brightly patterned with dark brown undulating bands bordered with bright yellow tubercles (Fig. 4C).

**Figure 6:**
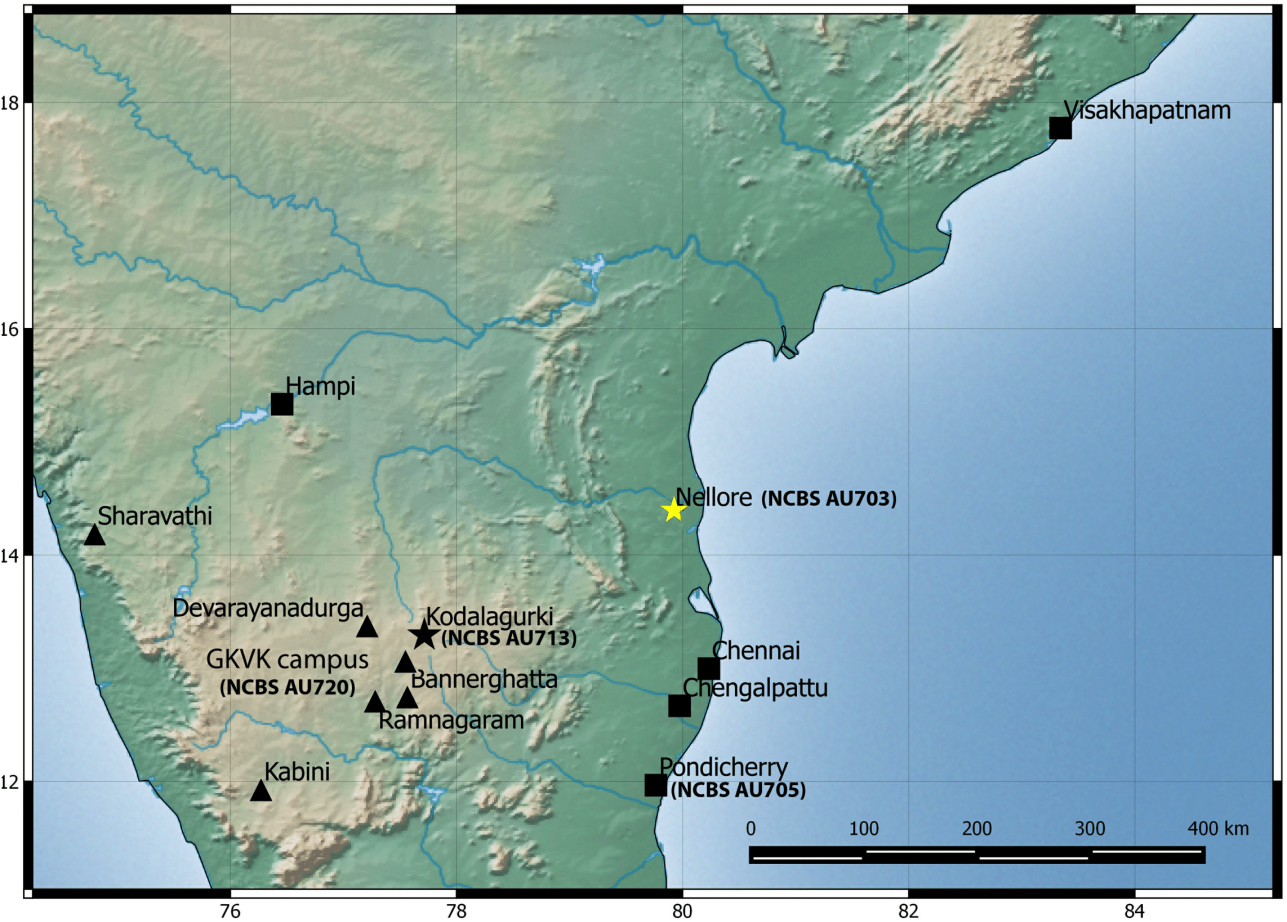
Map of peninsular India and northern Sri Lanka. Know localities are shown for *H. triedrus* (Yellow star & black squares) and *H. whitakeri* sp. nov., (black star and black triangles). A star highlights type localities.

*Natural history*: A species generally associated with termite mounds. Several individuals of different age classes can be seen occupying a single mound. Individuals can be seen at the entrance of the openings of termite mounds just after dusk and will retreat in the mound with the slightest disturbance. Juveniles when disturbed will attain a posture with their bodies high and the tail is moved slowly in a curling and uncurling manner, likely to draw attention toward the tail. Several individuals were found in a single mound when the termite mound was dug, along with scorpions of the genus *Heterometrus* sp., and frogs of the genus *Uperodon* sp. Individuals also seek shelter under boulders, abandoned houses during the day emerging just after dusk. Mostly terrestrial in its habits but will climb trees too. Breeding likely takes places from February to May as hatchlings and eggs have been seen in the months of April–May in Pondicherry and Kanyakumari. Occupies areas that are dry like scrub, dominated with boulders and even in cities closer to the coast. Widely distributed from Visakhapatnam in north to Kanyakumari in south and recorded from elevation ranging from 17 to 1,913 m AMSL. Recorded from the following states/Union territories in India: Kerala, Tamil Nadu, Andhra Pradesh and Pondicherry (Fig. 6).

*Suggested common name:* Southern termite hill gecko.

***Hemidactylus whitakeri* sp. nov.**
*Hemidactylus subtriedrus* Bauer et al., 2010
*Hemidactylus triedrus* Bansal & Karanth, 2010
Figs. 7 and 8; Table 1.

urn:lsid:zoobank.org:act:EDE27F23-187B-475F-BDB7-58F2016EDB5B.

**Figure 7:**
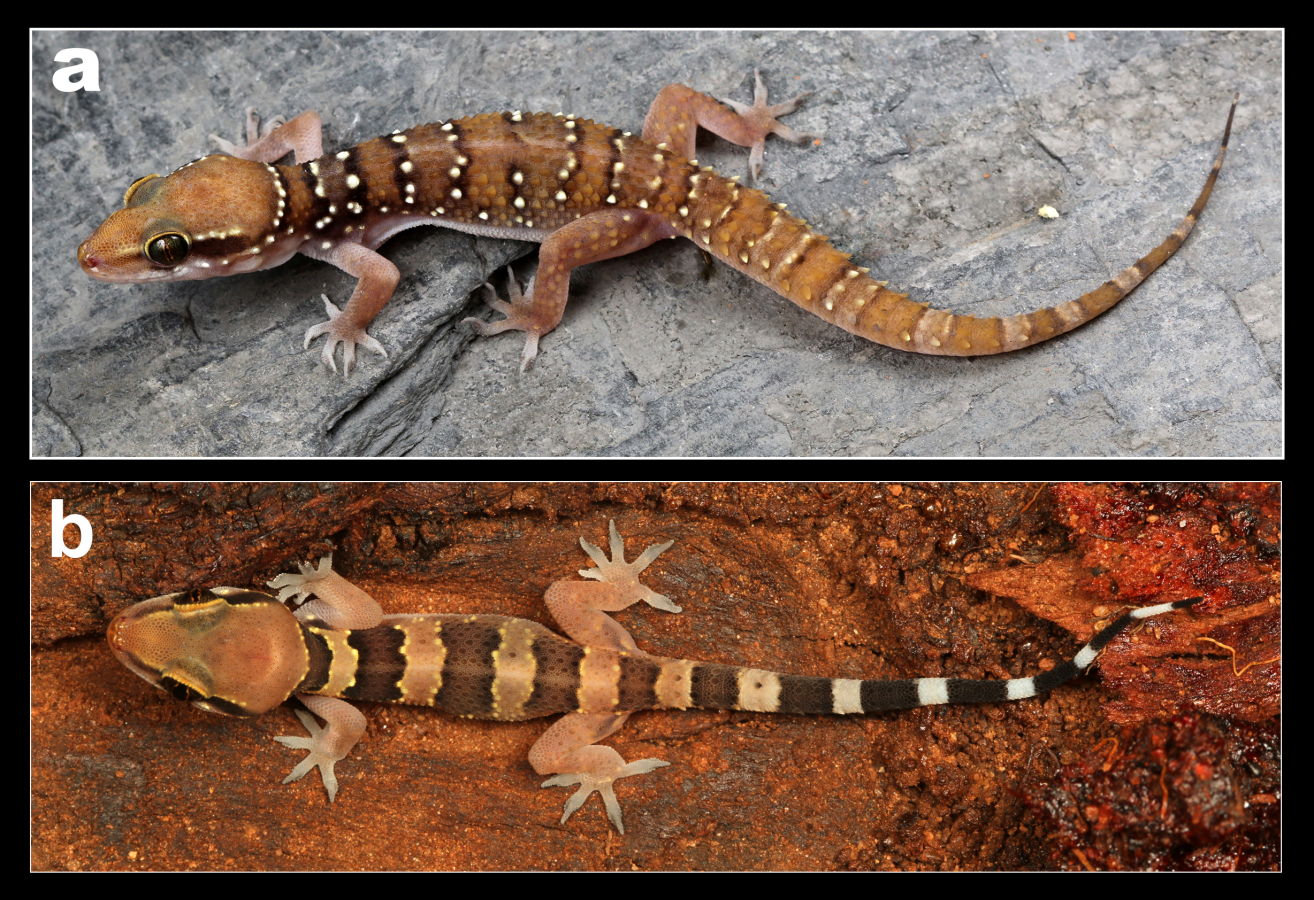
Coloration in life of *H. whitakeri* sp. nov. (A) Holotype female NCBS AU712, (B) uncollected juvenile from Bangalore.

**Figure 8:**
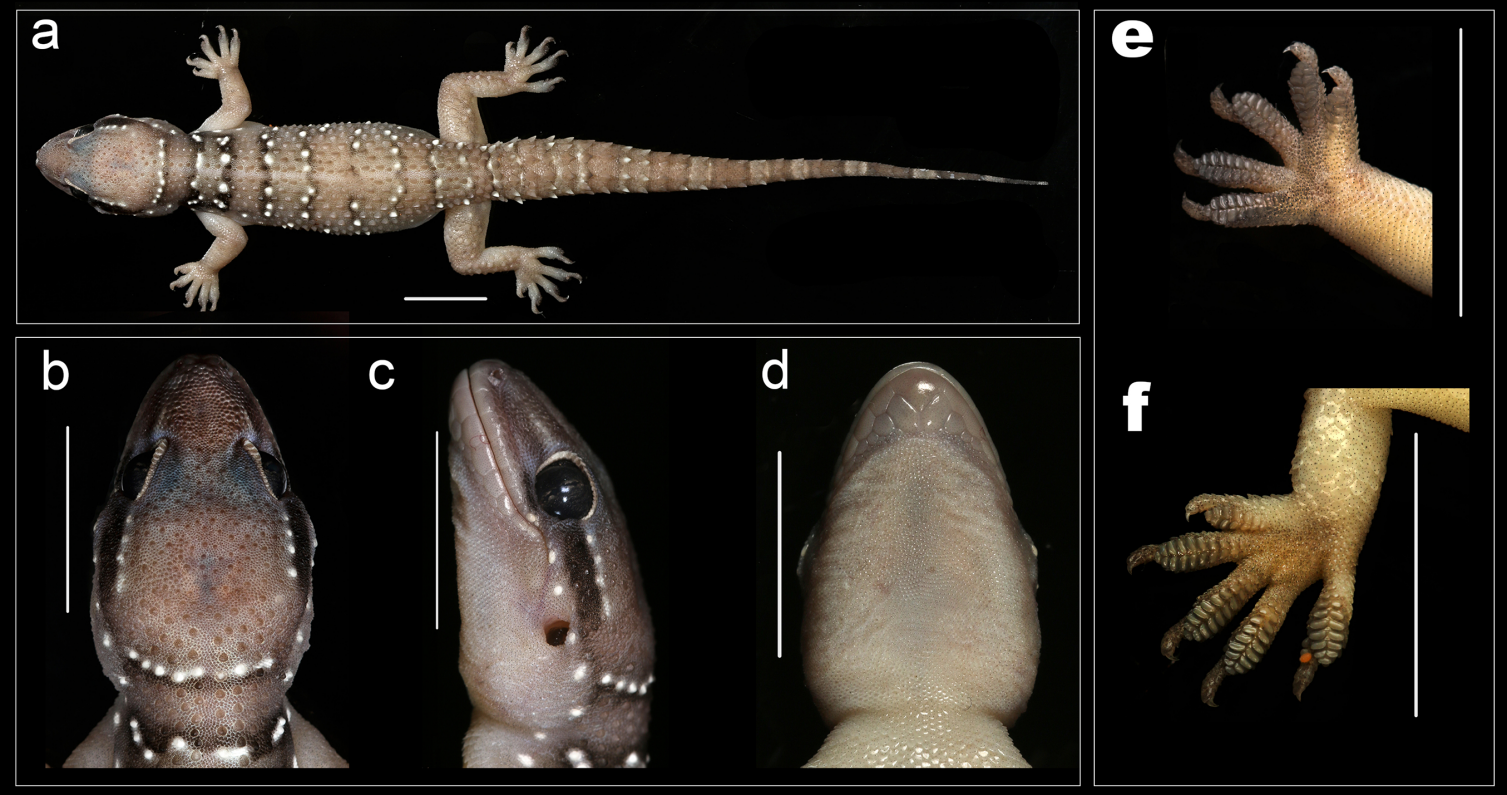
Female holotype *H. whitakeri* sp. nov. NCBS AU712. (A) Dorsal aspect, (B) dorsal aspect of head, (C) lateral aspect of head, (D) ventral aspect of head, (E) right manus, (F) right pes. Scale bar 10 mm.

*Holotype*: female NCBS AU712, near Kodalagurki village, Bangalore rural district, Karnataka, India (13.297508°N, 77.700259°E, elevation 950 m), collected by Mayuresh Ambekar & Gaurang Gowande on November 20th, 2017.

*Paratypes*: female NCBS AU713 same locality as holotype, collected by Rishikesh Patil and Zeeshan Mirza; two males NCBS AU719 & AU720, GKVK campus, Bangalore Karnataka, India (13.072962°N, 77.581215°E, elevation 932 m), collected by Akshay Khandekar on April 16th, 2018.

*Referred material*: female BNHS HTK1/2010 & male BNHS HTK2/2015 Kolli hills, Namakkal district, Tamil Nadu; male BNHS 1253 Nilgiri, Tamil Nadu; female BNHS 1827, two males ZSI 5852 & ZSI 5853 Bangalore, Karnataka.

*Diagnosis*: A medium sized fairly stout gecko, adults ranging 45–60 mm in SVL. Dorsum in a shade of light brown with paired, thin black edged white bands at regular intervals. Dorsal scalation on trunk granular, intermixed with enlarged, keeled 16–17 sub-trihedral tubercle rows arranged in fairly regular longitudinal series on dorsum. Seven lamellae (rarely six) under digit I of pes and manus, eight to nine under digit four of manus and pes. An angular series of seven to eight precloacal femoral pores separated at a mid-pelvic by a diastema of three non-pored scales.

*Hemidactylus whitakeri* sp. nov. differs from most congeners in bearing the following set of differing and non-overlapping characters: dorsum with large, keeled sub-trihedral tubercles in 16–17 fairly regular longitudinal rows (vs. few smooth or rounded tubercles in *H. aquilonius* McMahan & Zug, *H. flaviviridis* Rüppell, *H. frenatus* Schlegel, *H. garnotii* Duméril & Bibron, *H. leschenaultii* Duméril & Bibron, *H. giganteus* Stoliczka, *H. gujaratensis* Giri, Bauer, Vyas & Patil, *H. platyurus* Schneider, *H. anamallensis* Günther, *H. aaronbaueri* Giri, *H. yajurvedi* Murthy, Bauer, Lajmi, Agarwal & Giri, *H. hemchandrai* Dandge & Tiple); dorsal pattern with fairly distinct bands and dorsal tubercles sub-trihedral (vs. dorsal pattern with spots in *H. prashadi*); SVL 45–60 mm (vs. SVL <50 mm in *H. sataraensis* Giri & Bauer, *H. gracilis* Blanford, *H. reticulatus* Beddome, *H. albofasciatus* Grandison & Soman, *H. scabriceps* Annandale); SVL >80 mm *H. maculatus* Duméril & Bibron, *H. graniticolus* Agarwal, Bauer & Giri, *H. kangerensis*, *H. sushilduttai*, *H. vanam* Chaitanya, Lajmi & Giri, *H. acanthopholis* Mirza & Sanap, and *H. hunae* Deraniyagala; dorsum in a shade of brown with distinct, regularly spaced transverse bands on dorsum (vs. overall in a shade of brown to grey with dark spots, lacking distinct bands on dorsum in *H. persicus* Anderson, *H. robustus* Heyden, *H. turcicus* Linnaeus, *H. chipkali* Mirza & Raju, *H. treutleri* Mahony, *H. gleadowi* Murray, *H. parvimaculatus* Deraniyagala, *H. kushmorensis* Murray, *H. murrayi* Gleadow, *H. malcolmsmithi* Constable). The new species is most similar to *H. triedrus* in general appearance, however, differs in bearing 16–17 rows of keeled, sub-trihedral tubercles in fairly longitudinal rows (vs. 19–20 keeled, trihedral tubercles in *H. triedrus,* 15–16 trihedral tubercles in *H. sahgali* sp. nov.), bands on dorsum thin, paired and usually broken incomplete (vs. bands complete in *H. sahgali* sp. nov.), 7–8 precloacal femoral pores separated by a diastema of three non-pored scales (vs. 11–15 precloacal femoral pores separated by a diastema of 1–3 non-pored scales in *H. sahgali* sp. nov.). Postorbitofrontal slender in anteriorly, gradually widens posterior to the Fronto-Parietal suture as seen in *H. sahgali* sp. nov. (vs. Postorbitofrontal uniform in its wide throughout in *H. triedrus*), Frontal much wider posteriorly as in *H. sahgali* sp. nov. (vs. frontal narrow in *H. triedrus*); quadrate bone moderately robust (vs. quadrate bone robust and thin in *H. triedrus*, slender and arched in *H. sahgali* sp. nov.), surangular slender as in *H. triedrus* (vs. robust in its width lacking a distinct constriction at the suture between—articular surface and retroarticular process).

*Genetic divergence*: Genetic divergence within *H. whitakeri* sp. nov is 1–2% for the gene *cyt b* and 0–6% for *ND2*, divergence from *H. sahgali* sp. nov. and *H. triedrus* is 10–11% and 14–16% respectively for the gene *cyt* b. Divergence from *H. sahgali* sp. nov. and *H. triedrus* for the gene *ND*2 is 13–14% and 17–18% respectively.

*Etymology*: The specific epithet is a patronym honoring Romulus Earl Whitaker for his valuable contribution toward the study and conservation of reptiles of India.

*Description of female holotype NCBS AU712,* Figs. 7 and 8*:* The specimen is in good condition, preserved in a linear manner lacking the entire tail (Fig. 8A).

A medium sized gecko (SVL 53.7 mm) with a fairly large head (HL/SVL ratio 0.23), head slightly longer than wide (HW/HL ratio 0.88), head depressed (HH/HL ratio 0.61), distinct from neck (Figs. 8B–8D); canthus rostralis slightly inflated; snout short (SE/HW ratio 0.61), obtusely pointed from dorsal view and acutely in lateral view (Figs. 8B and 8C); longer than eye diameter (OD/SE ratio 0.48); scales on the snout sub-equal, convex, those anterior to the eye and on canthus rostralis, larger than the surrounding scales; eyes large (OD/HL ratio 0.25), pupil vertical with crenulated edges; supraciliaries larger on the anterior edge of the orbit, gradually decreasing in size as they progress toward the posterior portion of the orbit; ear-opening large, sub-oval, obliquely oriented, length less than half of the orbital diameter (EL/OD ratio 0.26) lobules absent; eye to ear distance greater than diameter of eye (EE/OD ratio 1.65); rostral quadrangle, much wider than deep, divided by a median suture for its entire length; rostral in contact with nasal, first supralabial and internasals; two large and a slightly smaller internasal between nasals; mental triangular, slightly wider (2.6) than long (2.8); two pairs of postmentals, anterior postmental longer (2.0) than wide (1.6); posterior pair of postmental smaller than anterior pair, nearly as long (1.1) as wide (1.0); anterior postmental in contact with mental, infralabial one and posterior pair of postmental; posterior postmentals less than half the size of the anterior one; anterior postmental nearly half the width to the first infralabial; posterior postmental half the width of second infralabials; posterior postmental in contact with anterior postmental, second and third infralabial; posterior borders of postmentals not aligned in a straight line (Fig. 8D); scales on throat and posterior region of the postmentals circular, smaller than the ones ventral aspect of trunk; supralabials (to midorbital position) six on left and right side; supralabials (to angle of jaw) eight on left and nine on right side; infralabials (to angle of jaw) seven on either sides.

Body elongate (TRL/SVL ratio 0.50) and dorsoventrally flattened; lacking distinct ventrolateral furrow; dorsal scalation on trunk granular intermixed with enlarged, keeled, sub-trihedral tubercles, fairly arranged in 16–17 longitudinal rows; dorsal tubercles on mid-dorsum situated in close proximity, slightly depressed, slightly longer (1.0) than wide (0.8); tubercles on the lateral aspect of the trunk larger, slightly spaced out in comparison with tubercles on mid-dorsum; ventral scales on trunk smooth, flat, larger than dorsal scales; mid body scales across belly 28–30.

Limbs moderately long, stout; digits dilated, bearing horizontally oriented lamellae on ventral surface; lamellae on basal half of digit I of manus and pes undivided, lamellae on rest other digits divided (excluding terminal lamellae); clawed, claw slightly smaller than length of the lamellar region; forelimbs short (FL/SVL ratio 0.15), slightly shorter than hind limbs (CL/SVL ratio 0.14). Terminal phalanx of all digits curved, arising angularly from distal portion of expanded lamellar pad, free portion of phalanx of all digits half to more than half long as the dilated portion. Lamellae beneath the digits, left manus 7-9-9-9-9 (Fig. 8E), right manus 7-9-9-9-9; left pes 7-10-10-9-8 (Fig. 8F), right pes 7-10-10-9-8. Lamellae not reaching the base of the digit IV of pes, covering 80% on the digit. Relative lengths of digits: III>V>IV>II>I (left manus), V>II>IV>III>I (left pes).

Tail moderately depressed, oval in cross section, longer than SVL (TL/SVL ratio 1.21). Caudal segments distinct; pholidosis of original tail dorsum with small, juxtaposed scales intermixed with large keeled trihedral tubercles, scales on regenerated portion of tail homogenous, slightly smaller compared to the scales on original tail and lacking tubercles. First tail segment with a whorl of eight large conical, keeled tubercles, second segment onward, each segment with six tubercles. Ventral aspect with large, broad scales covering about ∼60% of the tail width from base of tail to the tip. A single rounded post cloacal spur.

*Variation observed in examined specimens:* All examined specimens match the holotype except for morphometric data presented in Table 1. Specimens range in SVL from 45–60 mm. Pre-cloacal femoral pores range from seven to eight separated medially by three non-pored scales in males. The number of post cloacal spur in two in paratype male NCBS AU719 vs. one in NCBS AU720. Coloration in life is almost identical to *H. triedrus* (Figs. 7A and 7B).

*Natural history:* The types were found moving actively at a quarry site around 20:30 h. The holotype female contains two eggs in her body cavity suggesting that this species breeds during the months of November. Several hatchlings of the new species were encountered at Kengiri near Bangalore in the month of April. Similar in its habits to *H. triedrus* and can be seen on termite mounds. Its distribution is not well known and known from Bangalore in the state of Karnataka and the Nilgiri district in Tamil Nadu (Fig. 6).

*Suggested common name:* Whitaker’s termite hill gecko.

***Hemidactylus sahgali* sp. nov.**
*Hemidactylus triedrus* Boulenger, 1885:133 (in part); Smith, 1935:88 (in part); Minton, 1966:85; Bauer et al., 2010
Figs. 9 and 10; Table 2; Table S4.

urn:lsid:zoobank.org:act:E95E144A-4AAB-4AE3-9997-742FE0605F33

**Figure 9:**
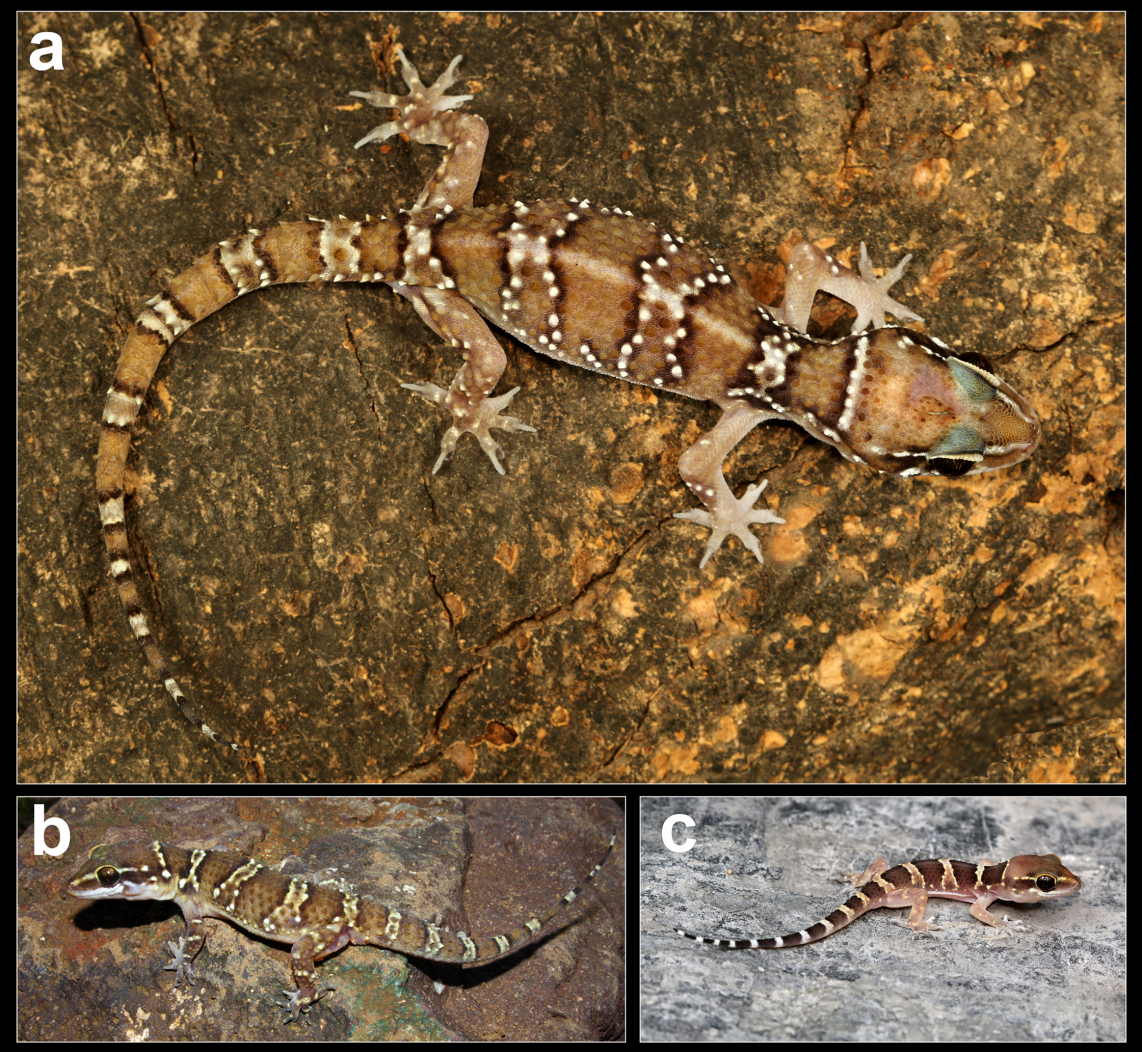
Coloration in life of *H. sahgali* sp. nov. (A) Paratype female NCBS AU709, (B) an uncollected male from Gautala Wildlife Sanctuary, Maharashtra, (C) uncollected juvenile from Pune.

**Figure 10:**
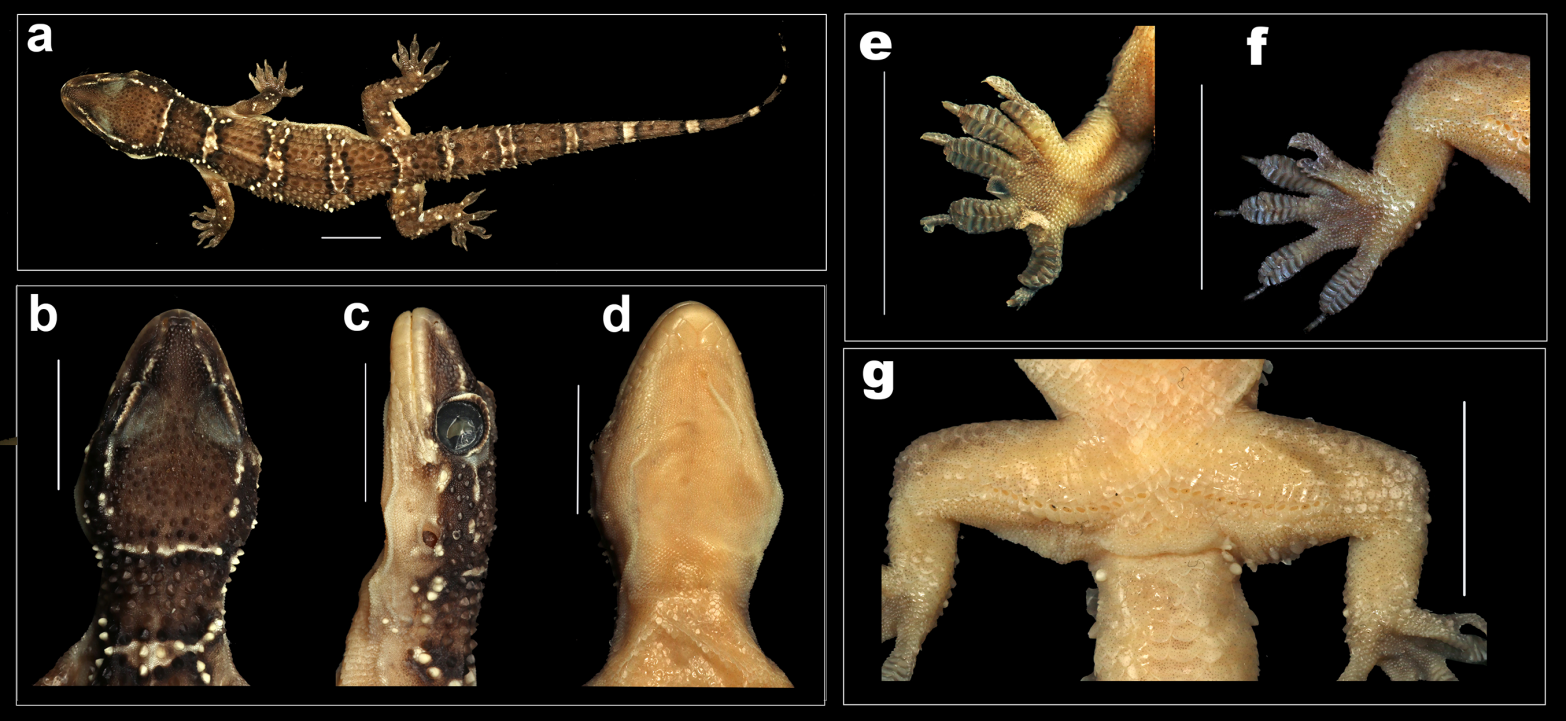
Male holotype *H. sahgali* sp. nov. NCBS AU708. (A) Dorsal aspect, (B) dorsal aspect of head, (C) lateral aspect of head, (D) ventral aspect of head, (E) right manus, (F) right pes, (G) cloacal region showing pre-cloacal femoral pores. Scale bar 10 mm.

**Table 2:** Morphological and meristic data for type specimens of *Hemidactylus sahgali* sp. nov.

Specimens number	NCBS AU708	NCBS AU709	BNHS 2496	BNHS 2497	BNHS 2498	BNHS 2499
	Holotype	Paratype	Paratype	Paratype	Paratype	Paratype
Sex	♂	♀	♂	♀	♀	♀
SVL	66.4	56.3	78.3	59.0	59.7	73.4
TRL	29.4	31.2	31.5	23.9	24.1	29.5
BW	14.2	12.6	17.8	14.2	13.1	13.9
CL	9.6	6.6	12.7	8.7	9.5	11.4
TL	76.3	72.3	51.27*	74.1	72.4	47.33*
TW	7.3	6.4	9.5	5.9	7.2	5.5
HL	14.4	16.2	23.4	19.0	17.7	21.6
HW	14.1	13.1	17.3	13.7	12.6	14.2
HH	8.4	9.6	9.2	7.3	6.3	7.6
FL	9.5	6.6	12.9	9.0	9.8	9.9
OD	4.1	4.0	5.3	4.4	4.2	5.0
NE	5.7	4.7	8.0	5.9	5.8	6.6
SE	8.3	7.6	9.6	7.8	7.4	8.9
EE	6.4	5.4	6.7	5.7	5.3	6.1
EL	1.5	1.3	2.7	2.2	2.0	2.6
IN	1.9	2.4	2.5	2.4	2.1	2.3
IO	8.0	5.7	9.7	7.9	7.6	7.8
Pores L	14	–	15	–	–	–
Pores R	13	–	15	–	–	–
gap btw pores	1	–	1	–	–	–
**Lamellae**						
L manus	8-9-9-9-8	7-8-9-8-8	7-9-8-9-9	8-9-10-9-9	8-9-10-9-9	9-10-9-10-10
R manus	8-9-9-9-8	7-8-9-8-8	7-9-9-9-9	8-9-10-9-9	8-9-9-9-9	8-9-10-10-9
L pes	8-9-10-10-9	7-9-9-8-8	8-10-9-9-9	8-10-10-10-9	8-10-10-9-9	7-10-10-10-10
R pes	8-10-11-9-9	7-9-9-8-8	8-10-10-9-9	8-10-10-10-9	8-10-10-9-9	8-9-10-9-9
Supralabials L/R	8/8	7/7	8/8	8/8	8/9	8/8
Infralabials L/R	8/8	7/8	7/7	8/7	7/7	7/7

**Note:**

* Indicates broken or regenerated tail.

*Holotype*: male, NCBS AU708, Khambha village, Visavadar, Junagadh district, Gujarat, India (21.283596°N, 70.640201°E, elevation 154 m), collected by Pranav Vaghashiya on May 29, 2017.

*Paratypes*: male BNHS 2496 Bilimora near Chikili, Navsari district, Gujarat (20.774864°N, 72.980325°E, elevation 15 m) collected by Harshil Patel, Kaushal Patel & Vaibhav Naik on June 3, 2017; female BNHS 2497 Ambapani Village, Tapi district, Gujarat (20.715000°N, 73.433046°E, elevation 208 m) collected by Harshil Patel & Vaibhav Naik on October 23, 2016; female BNHS 2498 Kangvai Village, Navsari district, Gujarat (20.870295°N, 73.173764°E, elevation 40 m) collected by Harshil Patel & Parimal Patel on September 8, 2015; BNHS 2499, Bilimora, Navsari district, Gujarat (20.774864°N, 72.980325°E, elevation 15 m) collected by Harshil Patel, & Vaibhav Naik on May 14, 2017; female NCBS AU709, Saswad, Pune district, Maharashtra (18.360547°N, 74.016699°E, elevation 848 m), collected by Varun Vaze, Gaurang Gowande & Rishikesh Patil on June 2017.

*Material examined*: India – female CAS 9414, Bhopal; female BNHS 93 Indore, Madhya Pradesh; female CAS/SUR 9630 Bishrampur, Chhattisgarh; male BNHS 94 Dangs, Gujarat; female BNHS 96 Hingolgadh; female BNHS 1478 Toranmal fort, Nandurbar, Maharashtra; male BNHS 1564 Tuljapur, Osmanabad district, Maharashtra; three males NCBS AU710—AU712 & female NCBS AU7013 Lonavala, Pune District, Maharashtra; two males ZSI 21483 & 21486 Pune, Maharashtra. Pakistan: male CAS 101355 Karachi, Sindh province.

*Diagnosis*: A medium sized fairly stout gecko, adults ranging 56–78 mm in SVL. Dorsum in a shade of light brown with paired, broad black edged white bands at regular intervals. Dorsal scalation on trunk, granular, intermixed with enlarged, keeled 15–16 trihedral tubercle rows arranged in fairly regular longitudinal series. Seven to eight lamellae under digit I of pes and manus, 8–10 under digit four of manus and pes. An angular series of 11–15 precloacal femoral pores separated at a mid-pelvic by a diastema of one to three non-pored scales.

*Hemidactylus sahgali* sp. nov. differs from most congeners in bearing the following set of differing and non-overlapping characters: dorsum with large, keeled trihedral tubercles in 16–18 fairly regular longitudinal rows (vs. few smooth or rounded tubercles in *H. aquilonius*, *H. flaviviridis*, *H. frenatus*, *H. garnotii*, *H. leschenaultii*, *H. giganteus*, *H. gujaratensis*, *H. platyurus*, *H. anamallensis*, *H. aaronbaueri*, *H. yajurvedi*, *H. hemchandrai*); dorsal pattern with fairly distinct bands and dorsal tubercles trihedral (vs. dorsal pattern with spots and dorsal tubercles sub-trihedral in *H. prashadi*); SVL 56–78 mm (vs. SVL <50 mm in *H. sataraensis*, *H. gracilis*, *H. reticulatus*, *H. albofasciatus*, *H. scabriceps*; SVL >80 mm *H. maculatus*, *H. graniticolus*, *H. kangerensis*, *H. sushilduttai*, *H. vanam*, *H. acanthopholis*, and *H. hunae*; dorsum in a shade of brown with distinct, regularly spaced transverse bands on dorsum (vs. overall in a shade of brown to grey with dark spots, lacking distinct bands on dorsum in *H. persicus*, *H. robustus*, *H. turcicus*, *H. treutleri*, *H. chipkali*, *H. gleadowi*, *H. parvimaculatus*, *H. kushmorensis*, *H. murrayi*, *H. malcolmsmithi*). The new species is most similar to *H. triedrus* in general appearance, however, differs in bearing 15–16 rows of keeled, trihedral tubercle in fairly longitudinal rows (vs. 19–20 keeled, trihedral tubercles in *H. triedrus,* 16–17 sub-trihedral tubercles in *H. whitakeri* sp. nov.), broad bands on dorsum complete (vs. bands thin, paired and usually broken in?), 11–15 precloacal femoral pores separated by a diastema of one to three non-pored scales (vs. seven to nine precloacal femoral pores separated by a diastema of one to three non-pored scales in *H. triedrus* and *H. whitakeri* sp. nov.).

Postorbitofrontal slender in anteriorly, gradually widens posterior to the Fronto-Parietal suture as seen in *H. whitakeri* sp. nov. (vs. Postorbitofrontal uniform in its wide throughout in *H. triedrus*), Frontal much wider posteriorly as in *H. whitakeri* sp. nov. (vs. frontal narrow in *H. triedrus*); quadrate slender and arched (vs. quadrate bone moderately robust and thick in *H. triedrus* and *H. whitakeri* sp. nov.), surangular robust in its width lacking a distinct constriction at the suture between—articular surface and retroarticular process (vs. slender in *H. triedrus* and *H. whitakeri* sp. nov.).

*Genetic divergence:* Genetic divergence between populations of within *H. sahgali* sp. nov is 0–1% for *cyt b* as well as *ND2*, divergence from *H. whitakeri* sp. nov. and *H. triedrus* is 10–11% and 14% respectively for the gene *cyt* b; Divergence from *H. whitakeri* sp. nov. and *H. triedrus* for the gene *ND*2 is 13–14% and 17–18% respectively.

*Etymology:* The specific epithet is a patronym honoring Bittu Sahgal, Editor and founder of Sanctuary Asia magazine for his contribution toward conservation of wildlife.

*Description of holotype male NCBS AU708* (Fig. 10)*:* Holotype in good condition preserved in a linear manner with only posterior part of the tail slightly curved (Fig. 10A).

A large sized gecko (SVL 66.4 mm) with a fairly large head (HL/SVL ratio 0.22), head nearly as long as wide (HW/HL ratio 0.97), head depressed (HH/HL ratio 0.58), distinct from neck (Figs. 10B and 10C); canthus rostralis slightly inflated; snout short (SE/HW ratio 0.59), obtusely pointed from dorsal view and acutely in lateral view (Fig. 10B); longer than eye diameter (OD/SE ratio 0.48); scales on the snout subequal, convex, those anterior to the eye and on canthus rostralis, larger than the surrounding scales; eyes large (OD/HL ratio 0.28), pupil vertical with crenulated edges; supraciliaries larger on the anterior edge of the orbit, gradually decreasing in size as they progress toward the posterior portion of the orbit; ear-opening large, sub-oval, obliquely oriented, one-third the length of the orbital diameter (EL/OD ratio 0.35) lobules absent; eye to ear distance greater than diameter of eye (EE/OD ratio 1.56); rostral quadrangle, much wider than deep, divided by a median suture for its entire length; rostral in contact with nasal, first supralabial and internasals; two large and a slightly smaller internasal between nasals; mental triangular, wider (4.2) than long (2.6); two pairs of postmentals, anterior postmental longer (2) than wide (1.6); posterior pair of postmental much smaller than anterior pair, wider (0.8) than long (1.0); anterior postmental in contact with mental, first infralabial and posterior pair of postmental; posterior postmentals less than half the size of the anterior one; anterior postmental slightly smaller than the width to the first infralabial; posterior postmental less than half the width of second infralabials; posterior postmental in contact with anterior postmental, first as well as second infralabial, posterior postmental exceeds the posterior border of anterior postmental (Fig. 10D); scales on throat and on the region posterior to postmentals circular, smaller than the ones ventral aspect of trunk; supralabials (to midorbital position) ten on left and right side; supralabials (to angle of jaw) twelve on left as well as right side; infralabials (to angle of jaw) nine on either sides.

Body elongate (TRL/SVL ratio 0.44) and dorsoventrally flattened; lacking distinct ventrolateral furrow; dorsal scalation on trunk granular intermixed with enlarged, keeled, trihedral tubercles, fairly arranged in 15–16 longitudinal rows; dorsal tubercles on mid-dorsum situated in close proximity, slightly depressed, slightly longer (1.4) than wide (1.3); tubercles on the lateral aspect of the trunk larger, slightly spaced out in comparison with tubercles on mid-dorsum; ventral scales on trunk smooth, flat, larger than dorsal scales; mid body scales across belly >30; 14 (left) and 13 (right) femoral pores separated medially by a single non-pored scale; non-pored scales slightly larger than pored scales (Fig. 10G).

Limbs moderately long, stout; digits dilated, bearing horizontally oriented lamellae on ventral surface; lamellae on basal half of digit I of manus and pes undivided, lamellae on rest other digits divided (excluding terminal lamellae); clawed, claw slightly smaller than length of the lamellar region; forelimbs short (FL/SVL ratio 0.14), equal in length with the hind limbs (CL/SVL ratio 0.14). Terminal phalanx of all digits curved, arising angularly from distal portion of expanded lamellar pad, free portion of phalanx of all digits half to more than half long as the dilated portion. Lamellae beneath the digits, left manus 8-9-9-9-8, right manus 8-9-9-9-8 (Fig. 10E); left pes 8-9-10-10-9, right pes 8-10-11-9-9 (Fig. 10F). Lamellae not reaching the base of the digit IV of pes, covering 80% on the digit. Relative lengths of digits: III>V>IV>II>I (left manus), V>II>IV>III>I (left pes).

Tail moderately depressed, oval in cross section, longer than SVL (TL/SVL ratio 1.12). Caudal segments distinct; pholidosis of original tail dorsum with small, juxtaposed scales intermixed with large keeled trihedral tubercles, scales on regenerated portion of tail homogenous, slightly smaller compared to the scales on original tail and lacking tubercles. First tail segment with a whorl of eight large conical, keeled tubercles, second segment onward, each segment with six tubercles. Ventral aspect with large, broad scales covering about ∼60% of the tail width from base of tail to the tip. One sub-conical post cloacal spur.

*Coloration in preservative:* Dorsum in a shade of dark brown with broad, fairly equidistant bands commencing at the nape to the tip of the tail. A total of five bands present, first narrow band on the nape, second on the intersection of the forelimbs, two on the trunk and the last one on the intersection of the hindlimbs. The bands are off-white with a diffused brown blotches, edged with black on its anterior and posterior ends. Limbs in the same color as the dorsum with a few white tubercles. A faint yellow stripe from the canthus rostralis through the supraciliary scales up till the temporal region. Ventrally off-white with each scale bearing sparse black speckles.

*Coloration in life:* Head, trunk and tail in a shade of brown, trunk coloration darker. Bands on the dorsum dark black edged with yellow borders on the head and trunk. Yellow edging of the bands absent on tail. Legs in a shade of light brown with a few scattered yellow spots, especially on tubercles. A yellowish stripe runs from mid canthus rostralis through the supraciliary scales up till the supra-tympanic region. Ventrally pinkish white.

*Variation observed in examined specimens:* All examined specimens match the holotype except for morphometric data presented in Table 2 and Table S4. Specimens range in SVL from 56–78 mm. Pre-cloacal femoral pores range from 11 to 15 separated medially by one to three non-pored scales in males. Dorsal tubercle rows vary in number ranging from 16 to 18.

*Natural history:* A species associated with termite mounds as its related species *H. triedrus*. Found in dry open scrub areas with boulders. Seen actively moving about on the ground just after dusk. Hatchlings have been seen in the month of May. Distributed throughout the Deccan Traps, its distribution extends beyond the traps north-west into Pakistan. In India, it is recorded from the following states: Maharashtra, Gujarat, Madhya Pradesh, Rajasthan and Chhattisgarh records ranging in elevation from 11 to 1,191 m AMSL. All records of the species from Pakistan are from elevation ranging from 15 to 25 m AMSL.

*Suggested common name:* Sahgal’s termite hill gecko.

## Discussion and Conclusion

**Figure 11:**
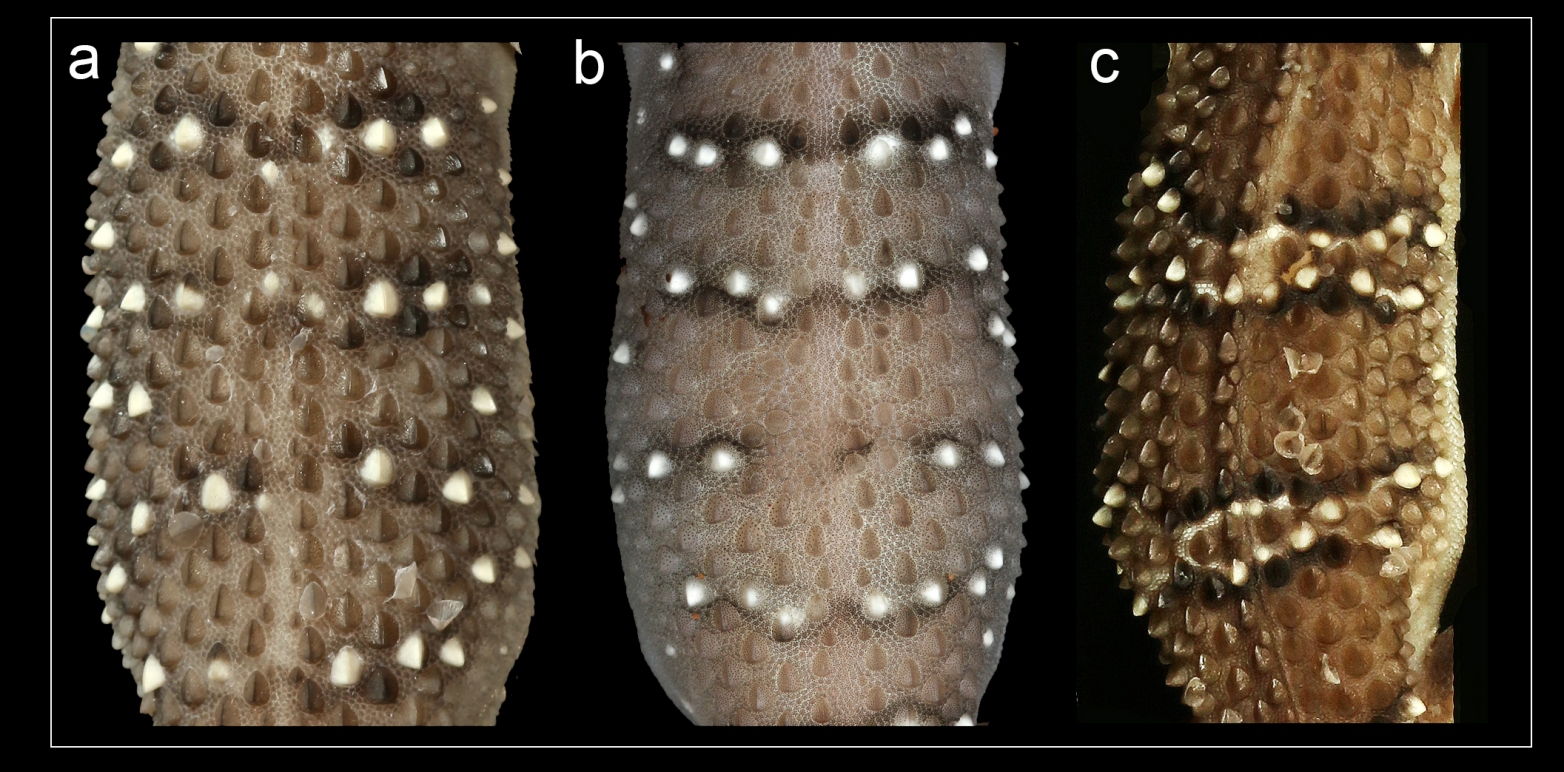
Mid-dorsal trunk tubercle rows. (A) *H. triedrus* NCBS AU703, (B) holotype *H. whitakeri* sp. nov. NCBS AU712, (C) holotype *H. sahgali* sp. nov. NCBS AU708.

**Figure 12:**
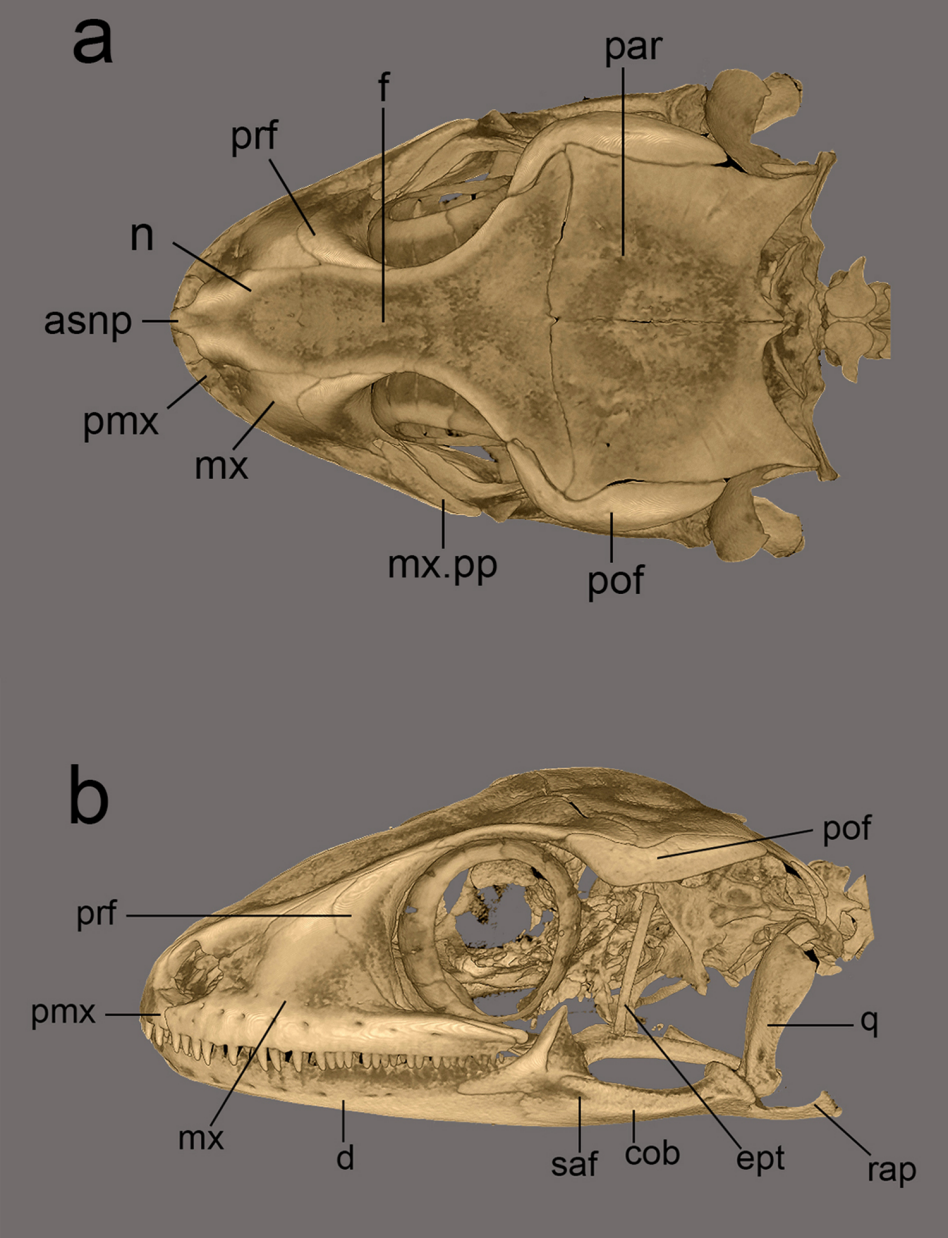
Micro-CT scan images of the skull of male paratype NCBS AU719. *Hemidactylus whitakeri* sp. nov (A) dorsal, (B) lateral view. Study sites: *asnp*, ascending nasal process of premaxilla; *cob*, compound bone; *ept*, epipterygoid; *f,* frontal; *mx*, maxilla; *mx.pp*, posterior process of maxilla; *par*, parietal; *pmx*, premaxilla; *pof*, postorbitofrontal; *prf,* prefrontal; *q*, quadrate; *rap*, retroarticular process; *saf*, surangular foramen.

**Figure 13:**
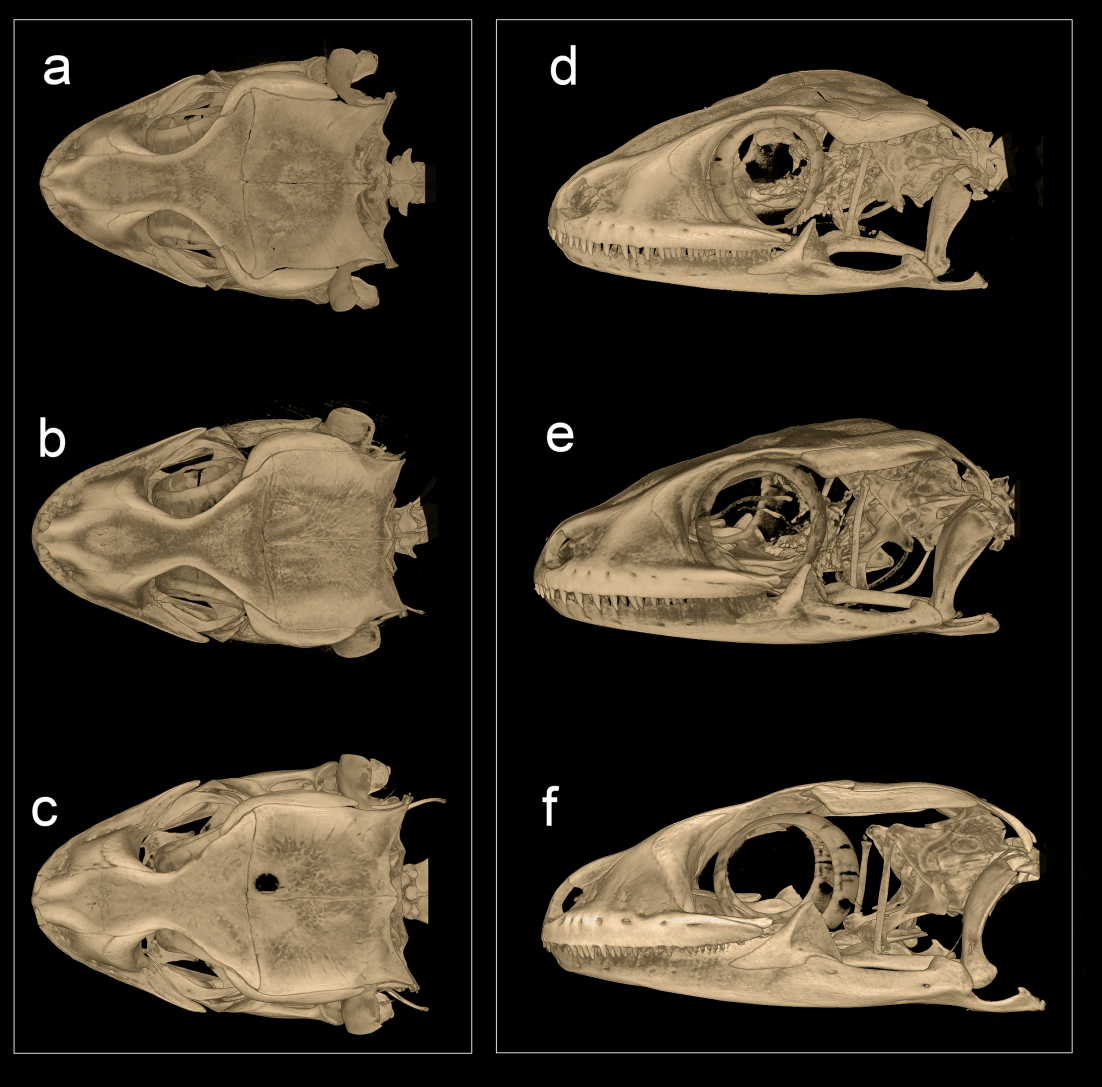
Micro-CT scan images of the skull of *Hemidactylus triedrus* species complex. (Dorsal & lateral view), (A) & (D) *H. whitakeri* sp. nov. paratype NCBS AU719, (B) & (E) *H. triedrus* NCBS AU705, (C) & (F) *H. sahgali* sp nov. NCBS AU710.

Phylogenetic relationships within *Hemidactylus* reveal a major endemic radiation in South Asia and largely concentrated in India and Sri Lanka (Bauer et al., 2010). The *H. prashadi* clade (Agarwal, Giri & Bauer, 2011; Mirza, Bhosale & Patil, 2017) contains several large members of the genus which are endemic to India and Sri Lanka which bear large keeled tubercles. Phylogenetic relationship within the *H. prashadi* clade are not well resolved likely due to incomplete taxon sampling. However, previous studies present evidence for high genetic divergence in some members of the clade, suggesting presence of cryptic species; a prime example of this is *H. triedrus* (Mirza, Bhosale & Patil, 2017). *H. triedrus* was considered to be widespread across India and Sri Lanka and parts of Pakistan despite that there were three distinct and diagnosable populations based on morphology alone. This taxonomic flux largely arose likely from the fact that most authors who revised this taxon did not examine the type specimen of *H. triedrus* and/or specimens that were used for molecular work, which led to description of *H. subtriedrus* and *H. lankae* as well as an unjustified elevation to a species rank from its sub-specific status of the latter two species by Bauer et al. (2010). The present study refutes the current taxonomy of *H. triedrus*, as a phylogenetic analysis based on a ∼288 bp of *cyt b* and a 966 bp of *ND2* shows that the sequence of *H. lankae* (Bauer et al., 2010) is embedded within a clade containing *H. triedrus* sensu stricto, two sequences of captive *H. triedrus* (Bauer et al., 2010) represent a new species, *H. sahgali* sp. nov. and three sequences of *H. triedrus* (Bansal & Karanth, 2010) plus *H. subtriedrus* (Bauer et al., 2010) represent *H. whitakeri* sp. nov. Morphologically, *H. whitakeri* sp. nov. shows affinity to *H. triedrus* in sharing similar markings, habitus, number of precloacal pores and lamellae. Tubercle shape is sub-trihedral in *H. whitakeri* sp. nov. and trihedral in *H. triedrus* and *H. sahgali* sp. nov. (Fig. 11). However, genetically *H. whitakeri* sp. nov. is sister to *H. sahgali* sp. nov. based on concatenated data comprising of *ND2, RAG1* and *PDC*, and *H. triedrus* is sister to this group (Fig. 2). *H. whitakeri* sp. nov. exhibits similar skull osteology features as seen in *H. sahgali* sp. nov. (Figs. 12 and 13).

**Figure 14:**
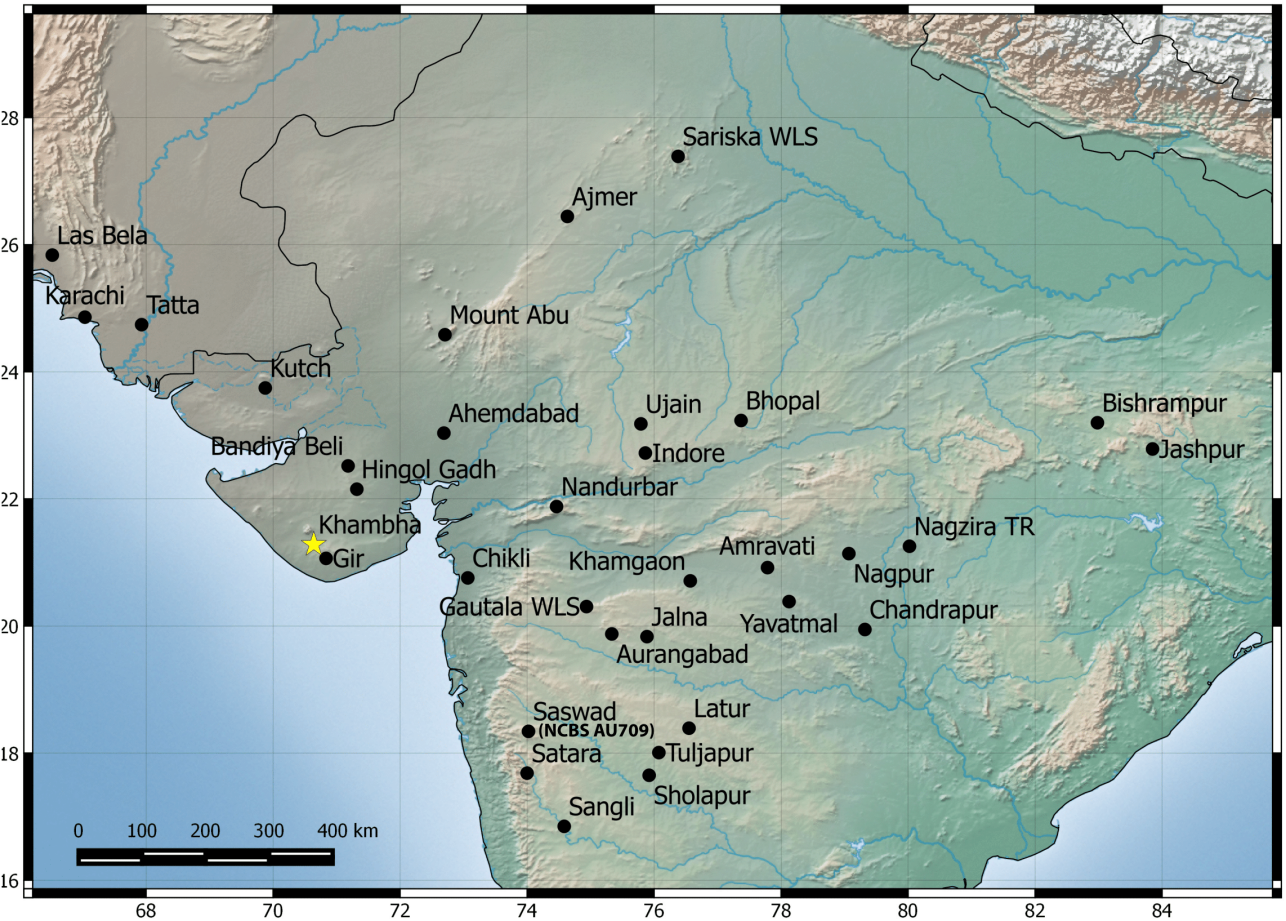
Map showing known distribution of *H. sahgali* sp. nov. based on examine specimens and photographic records. The yellow star highlights the type locality.

Among the three species, *H. sahgali* sp. nov. appears to be widespread across western-central India (Fig. 14) and shows very low sequence divergence suggesting a very recent colonization. The distribution of the other two sister species is not well known and these species are only known from a few localities. *H. triedrus* is known with certainty from coastal Andhra Pradesh, Pondicherry and Tamil Nadu, whereas *H. whitakeri* sp. nov. is known to occur in and around Bangalore and Bellary District of Karnataka and Nilgiri district in Tamil Nadu. The distribution of *H. triedrus* and *H. whitakeri* sp. nov. is not well known and further collection across southern India is necessary to define their distribution and their range boundaries.

The divergence dates estimated by previous work show that *H. sahgali* sp. nov. and *H. triedrus* shared a common ancestor about 11 (8–12) million years ago (Bauer et al., 2010; Agarwal & Karanth, 2015) which coincides with intensified aridification followed by onset of Indian monsoons. A common pattern of diversification that coincides with intensified aridification during the Late Miocene is observed across lizards in India (Agarwal & Ramakrishnan, 2017; Deepak & Karanth, 2017) and likely, it also appears to be a case with dry zone species of the genus *Hemidactylus* inhabiting Indian peninsula.

Among Sri Lankan species of the genus *Hemidactylus*, only two species, *H. depressus* and *H. pieresii* bear 15–19 and 17–20 pre-cloacal femoral pores respectively (Batuwita & Pethiyagoda, 2012). The range specified by Deraniyagala (1953) for *H. lankae* 13–19, falls within the range of *H. depressus* and *H. pieresii.* Until recently, *H. pieresii* was considered a synonym of *H. depressus* (Smith, 1935), and hence Deraniyagala (1953) could have attributed specimens of *H. depressus* + *H. pieresii* to his *H. lankae.* The identity of *H. lankae* remains unclear as no specimen examined by Deraniyagala (1953) is extant and it would be wise to treat it as *nomen dubium* until specimens that match the description are found.

Discovery of two new gecko species with such a wide distribution highlights the need for undertaking dedicated taxonomic revision of widespread taxa which likely represent multiple species. This situation parallels with most gekkonid lizards, example *H. maculatus* (Agarwal, Giri & Bauer, 2011; Javed et al., 2011; Mirza & Sanap, 2014; Giri et al., 2017; Mirza, Bhosale & Patil, 2017), *H. brookii* (Mahony, 2011; Lajmi, Giri & Karanth, 2016; Mirza & Raju, 2017) and *Cyrtodactylus* spp. (Agarwal et al., 2014, 2016). Many of these recently described species would not have received conservation attention they deserve and would have been considered Least Concern as per International Union for Conservation of Nature (IUCN) guidelines, had these not been described as a result of systematic investigations. Such a classification can be detrimental to any species as most funding agencies are biased toward threatened species and many species that deserve the funds and attention suffer. It is hoped that funding agencies have a neutral approach and provide funding to deserving projects irrespective of the IUCN conservation status alone.

A common practice for naturalist and even taxonomists during biodiversity surveys is to overlook subtle difference in commonly distributed species and attribute them to a readily available name when the species in question might not be what it has been considered conspecific with (Zug et al., 2006). This practice leads to unfortunate consequences of underrating a regions biodiversity, detrimental from the point of conservation of a particular landscape and/or species.

## Supplemental Information

10.7717/peerj.5341/supp-1 Supplemental Information 1 Fig. S1. ML based result from bPTP. Numbers at nodes and tips signify support to species.

10.7717/peerj.5341/supp-2 Supplemental Information 2 Fig. S2. Nucleotide sequence alignment for the gene *cyt b*. Note the 9bp indel from 281bp to 280bp. Alligned with a reference sequence of Gekko gekko.

10.7717/peerj.5341/supp-3 Supplemental Information 3 Fig. S3. ML tree for selected *Hemidactylus* spp. based on fragment of *cyt b* gene. Sequence substitution model GTR+G.

10.7717/peerj.5341/supp-4 Supplemental Information 4 Table S1. Accession numbers for sequences used in the present study. Numbers in bold show sequences generated in the present work. Names within brackets refers to the old taxonomy.

10.7717/peerj.5341/supp-5 Supplemental Information 5 Table S2. Best fit model for sequence evolution and partitioning scheme selected in PartitionFInder for assessing phylogenetic relationships using maximum likelihood in RAxML and Bayesian inference in MrBayes.

10.7717/peerj.5341/supp-6 Supplemental Information 6 Table S3. Morphological and meristic data for specimens of *Hemidactylus triedrus*. ‘*’ indicates broken or regenerated tail.

10.7717/peerj.5341/supp-7 Supplemental Information 7 Table S4. Morphological and meristic data for specimens of *Hemidactylus sahgali* sp. nov. ‘*’ indicates broken or regenerated tail.

10.7717/peerj.5341/supp-8 Supplemental Information 8 Table S5. Result of bPTP for ML support value for each species.

10.7717/peerj.5341/supp-9 Supplemental Information 9 Table S6. Un-corrected p-distance for selected Hemidactylus spp. for the gene cyt b.

10.7717/peerj.5341/supp-10 Supplemental Information 10 Table S7. Un-corrected p-distance for selected Hemidactylus spp. for the gene ND2.

10.7717/peerj.5341/supp-11 Supplemental Information 11 Table S8. Comparison of taxonomy of *H. triedrus* group since 2010.

## Additional Information and Declarations

### Field Study Permissions

The following information was supplied relating to field study approvals (i.e., approving body and any reference numbers): Specimens were captured and processed following the guidelines and protocols stated in the collecting permits (B/WPS/8/9388-92/2013-14, 71/16/4454 & 71/16/4487) and agreements obtained from the competent authorities.

### DNA Deposition

The following information was supplied regarding the deposition of DNA sequences: All DNA sequences are available at GenBank. The accession numbers are supplied in Table S1.

### Data Availability

The following information was supplied regarding data availability: All freshly collected specimens are deposited in the collection of the Bombay Natural History, Mumbai and Research collection of the National Centre for Biological Sciences, Bangalore. The raw figures and tables are available at Mirza, Zeeshan; Gowande, Gaurang G.; Patil, Rishikesh; Ambekar, Mayuresh; Patel, Harshil (2018): First appearance deceives many: disentangling the Hemidactylus triedrus species complex using an integrated approach. figshare. Fileset. DOI 10.6084/m9.figshare.6818135.v1.

### New Species Registration

The following information was supplied regarding the registration of a newly described species: Publication LSID: urn:lsid:zoobank.org:pub:28E6F879-6559-4CE3-9BE9-5A51F10EAD26. *Hemidactylus sahgali*: urn:lsid:zoobank.org:act:E95E144A-4AAB-4AE3-9997-742FE0605F33. *Hemidactylus whitakeri*: urn:lsid:zoobank.org:act:EDE27F23-187B-475F-BDB7-58F2016EDB5B.
