# Supplementary figures and images for: First appearance deceives many: disentangling the Hemidactylus triedrus species complex using an integrated approach
Source: PeerJ. 2018 Aug 2;6:e5341. doi: 10.7717/peerj.5341 (PMC6076986; doi:10.7717/peerj.5341)

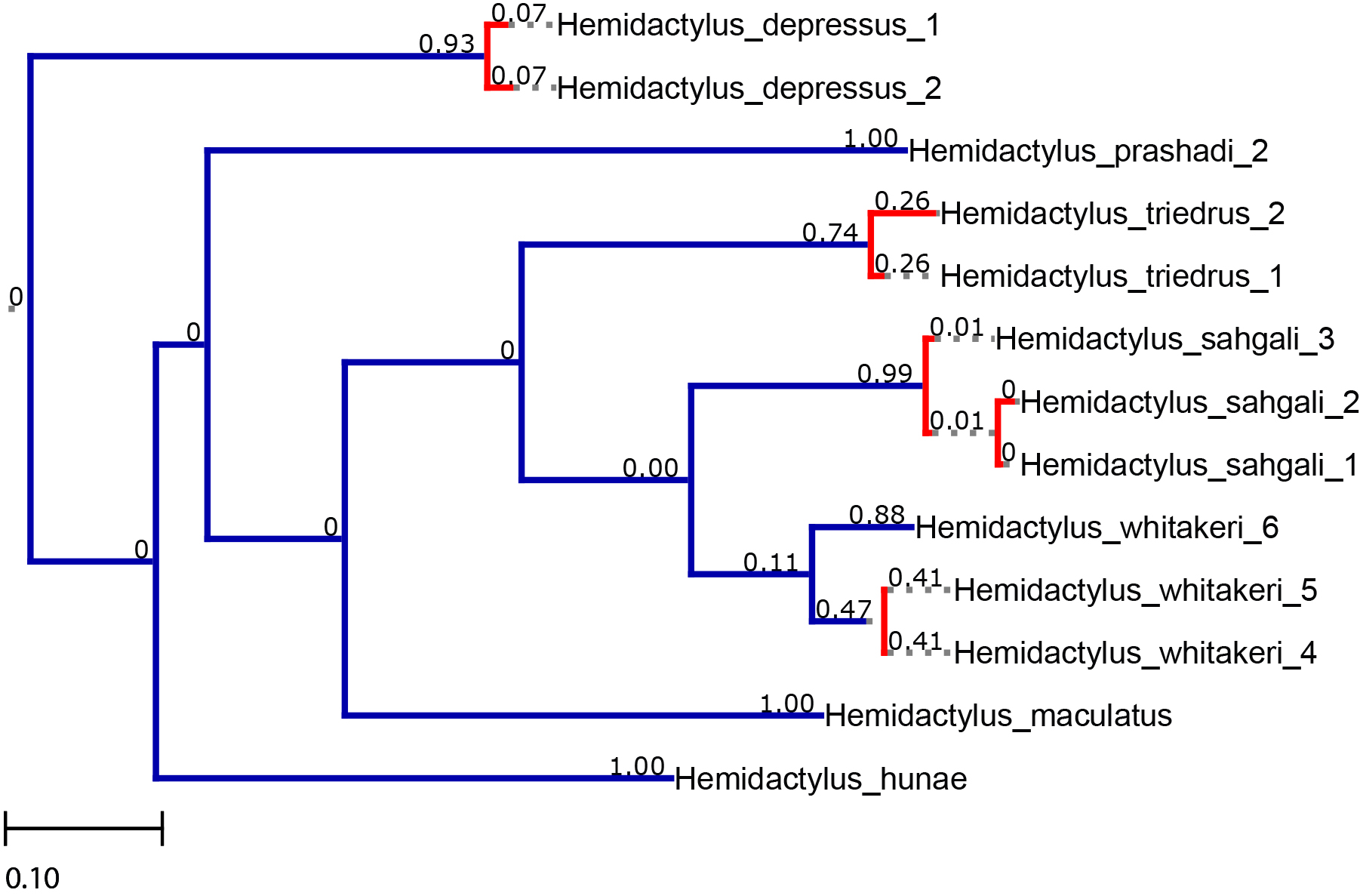

Supplement: Supplemental Information 1 — Numbers at nodes and tips signify support to species. [file peerj-06-5341-s001.jpg]

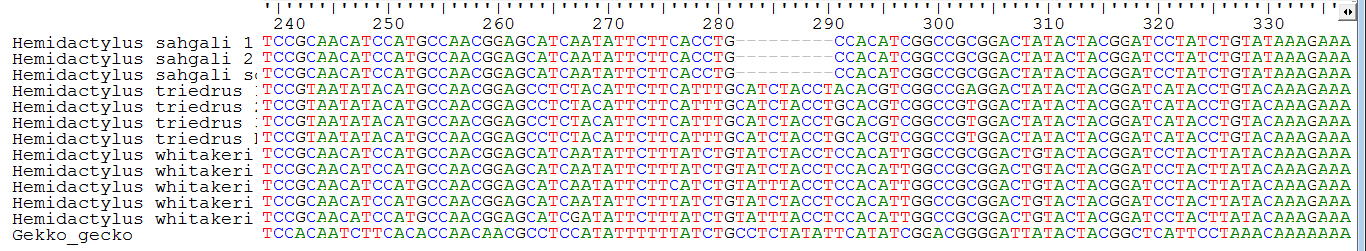

Supplement: Supplemental Information 2 — Note the 9bp indel from 281bp to 280bp. Alligned with a reference sequence of Gekko gekko. [file peerj-06-5341-s002.jpg]

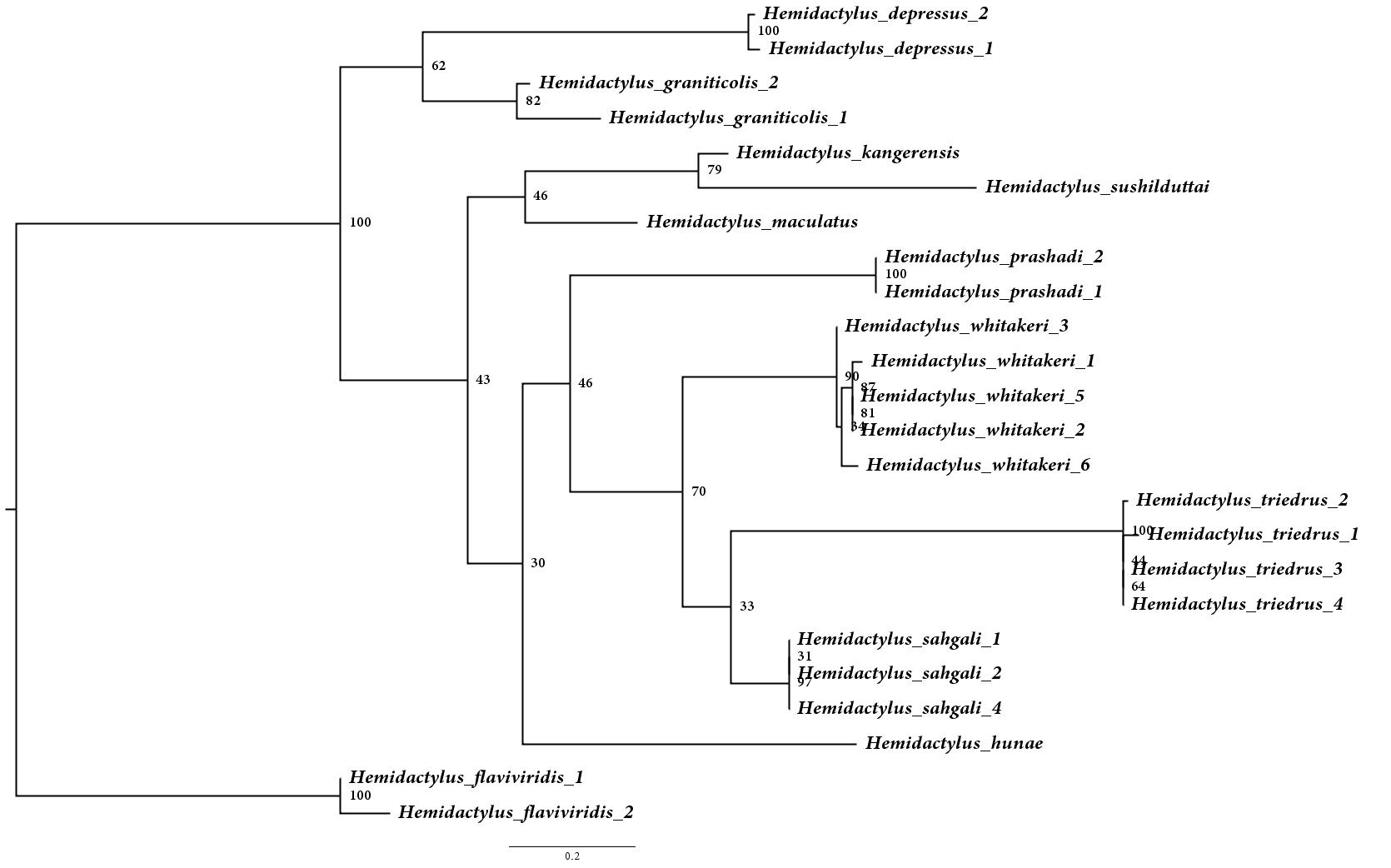

Supplement: Supplemental Information 3 [file peerj-06-5341-s003.jpeg]
