# Supplementary material for: First appearance deceives many: disentangling the Hemidactylus triedrus species complex using an integrated approach
Source: PeerJ. 2018 Aug 2;6:e5341. doi: 10.7717/peerj.5341 (PMC6076986; doi:10.7717/peerj.5341)
Supplement: Supplemental Information 4 — Numbers in bold show sequences generated in the present work. Names within brackets refers to the old taxonomy. [file peerj-06-5341-s004.docx]

| Species | *cyt* b | *ND2* | *Rag-1* | PDC | Specimen code | Locality | Reference |
| --- | --- | --- | --- | --- | --- | --- | --- |
| *H. acanthopholis 1* | MG711525 | MG711530 | MG711538 | MG711533 | CES 14009 | India, Tamil Nadu, Kallidaikurchi | Chaitnya et al. 2018 |
| *H. acanthopholis 2* | MG711526 | MG711531 | MG711539 | MG711534 | CES 17066 | India, Tamil Nadu, Kallidaikurchi | Chaitnya et al. 2018 |
| *H. depressus* 1 | HM559593 | HM559625 | HM559691 | - | AMB 7481 | Sri Lanka, Matale | Bauer et al. 2010 |
| *H. depressus* 2 | HM559591 | HM559623 | HM559689 | - | AMB 7440 | Sri Lanka, Dumbulayala | Bauer et al. 2010 |
| *H. flaviviridis* 1 | HM559595 | HM559627 | HM559693 | - | CAS 228540 | UAE, Dubai | Carranza & Arnold 2006 |
| *H. flaviviridis* 2 | EU268387 | EU268355 | EU268294 | - | FMNH 245515 | Pakistan, Punjab Province | Carranza & Arnold 2006 |
| *Hemidactylus hunae* | HM559606 | HM559640 | HM559706 | - | AMB 7416 | Sri Lanka, Pitakumbura | Bauer et al. 2010 |
| *Hemidactylus kangerensis* | KY938009 | - | - | - | BNHS 2486 | India, Chhattisgarh, Kangerghati National Park | Mirza et al. 2017 |
| *H. graniticolis 1* | HM595663 | - | - | - | CES/07/005 | India, Karnataka, Ramnagar | Bansal & Karanth 2010 |
| *H. graniticolis* 2 | HM595664 | - | HM622361 | - | CES/08/028 | India, Tamil Nadu, Nilgiri Hills | Bansal & Karanth 2010 |
| *Hemidactylus maculatus* | HM559607 | HM559641 | HM559707 | - | BNHS 1516 | India, Maharashtra, Zirad | Bauer et al. 2010 |
| *H. prashadi 1* | HM595668 | - | HM622364 | - | CES/07/040 | India, Karnataka, Castle Rock | Bauer et al. 2010 |
| *H. prashadi* 2 | HM559609 | HM559643 | HM559709 | - | JB 30 | India | Bauer et al. 2010 |
| *Hemidactylus triedrus 1*  *(H. lankae)* | HM559615 | HM559648 | HM559714 | - | AMB 7453 | Sri Lanka, nr. Medavachchiya | Bauer et al. 2010 |
| *Hemidactylus triedrus* 2 | **MG742360** | **MH666065** | - | - | NCBS AU703 | India, Andhra Pradesh, Nellore | Present work |
| *Hemidactylus triedrus* 3 | **MG742361** | **-** | - | - | NCBS AU705 | India, Pondicherry | Present work |
| *Hemidactylus triedrus* 4 | **MH666070** | **-** | - | - | NCBS XXXX | India, Karnataka, Hampi | Present work |
| *H. sahgali* 1 (*H. triedrus*) | HM559617 | HM559650 | HM559716 | **-** | JB 08 | Pakistan | Bauer et al. 2010 |
| *H. sahgali* 2 (*H. triedrus*) | HM559616 | HM559649 | HM559715 | - | JB 09 | India | Bauer et al. 2010 |
| *H. sahgali* 3 | - | **MG742362** | **-** | **-** | NCBS AU709 | India, Maharashtra, Saswad | Present work |
| *H. sahgali* 4 (*H. triedrus*) | MH454709 | MH454771 | MH454735 | - | CES 13191 | India, Maharashtra, Solapur | Chaitnya et al. 2018 |
| *Hemidactylus sushilduttai* | MF668228 | - | - | **-** | ESV 112 | Giri et al. 2017 |  |
| *H. vanam* 1 | MG711527 | MG711532 | MG711540 | MG711535 | BNHS 2329 | India, Tamil Nadu, Meghamalai | Chaitnya et al. 2018 |
| *H. vanam* 2 | MG711528 | - | MG711541 | MG711536 | ZSI/WGRC/IR/V .NO2635 | India, Tamil Nadu, Meghamalai | Chaitnya et al. 2018 |
| *H. whitakeri* 1 (*H. triedrus*) | HM595673 | - | HM622365 | - | CES/07/007 | India, Karnataka, Ramnagar | Bansal & Karanth 2010 |
| *H. whitakeri* 3 (*H. triedrus*) | HM595675 | - | - | **-** | CES/07/023 | India, Karnataka, Atigulipura | Bansal & Karanth 2010 |
| *H. whitakeri* 2 | **MG742359** | **MG742359X** | **-** | - | NCBS-AU713 | India, Karnataka, Kodalagurki | Present work |
| *H. whitakeri* 5 (*H. subtriedrus*) | **-** | HM627909 | - | - | JB | India | Bauer et al. 2010 |
| *H. whitakeri* 4 | **MH666068** | **MH666066** | - | - | NCBS-AU713 | India, Karnataka, Kodalagurki | Present work |
| *H. whitakeri* 6 | **MH666069** | **MH666067** | **-** | **-** | NCBS-AU720 | India, Karnataka, Bangalore | Present work |

Literature cited:

Bansal R., Karanth KP. 2010. Molecular Phylogenetics and Evolution Molecular phylogeny of *Hemidactylus* geckos (Squamata: Gekkonidae) of the Indian subcontinent reveals a unique Indian radiation and an Indian origin of Asian house geckos. *Molecular Phylogenetics and Evolution* 57:459–465. DOI: 10.1016/j.ympev.2010.06.008.

Bauer AM., Jackman TR., Greenbaum E., Giri VB., Silva A De. 2010. South Asia supports a major endemic radiation of *Hemidactylus* geckos. *Molecular Phylogenetics and Evolution* 57:343–352. DOI: 10.1016/j.ympev.2010.06.014.

Carranza, S., Arnold, E.N., 2006. Systematics, biogeography, and evolution of Hemidactylus geckos (Reptilia: Gekkonidae) elucidated using mitochondrial DNA sequences. Mol. Phylogenet. Evol. 38, 531–545.

Chaitanya, R., Lajmi, A. & Giri, V.B. (2018) A new cryptic, rupicolous species of Hemidactylus Oken, 1817 (Squamata:

Gekkonidae) from Meghamalai, Tamil Nadu, India. Zootaxa, 4374 (1), 49–70

.

Giri VB., Bauer AM., Mohapatra PP., Srinivasulu C., Agarwal I. 2017. A new species of large-bodied, tuberculate *Hemidactylus* Oken (Squamata: Gekkonidae) from the Eastern Ghats, India. *Zootaxa* 0000:1–15. DOI: 10.11646/zootaxa.4347.2.8.

Mirza ZA., Bhosale H., Patil R. 2017. A new large species of gecko of the genus *Hemidactylus* Oken, 1817 (Reptilia: Sauria: Gekkonidae) from the Eastern Ghats, India. *Comptes Rendus - Biologies* 340:531–540. DOI: 10.1016/j.crvi.2017.09.003.
