# Supplementary material for: First appearance deceives many: disentangling the Hemidactylus triedrus species complex using an integrated approach
Source: PeerJ. 2018 Aug 2;6:e5341. doi: 10.7717/peerj.5341 (PMC6076986; doi:10.7717/peerj.5341)
Supplement: Supplemental Information 5 [file peerj-06-5341-s005.docx]

Table S2: Best fit model for sequence evolution and partitioning scheme selected in PartitionFInder for assessing phylogenetic relationships using maximum likelihood in RAxML and Bayesian inference in MrBayes.

| **RAxML** | | **MrBayes** | |
| --- | --- | --- | --- |
| rag_pos1  pdc_pos2, pdc_pos3, rag_pos2  pdc_pos1, rag_pos3  nd_pos1  nd_pos2  nd_pos3 | GTR+I+G | rag_pos1  pdc_pos2, rag_pos2  rag_pos3  nd_pos1  nd_pos2  nd_pos3  pdc_pos1  pdc_pos3 | HKY+I  GTR+I  HKY+I+G  GTR+G  GTR+I+G  GTR+I+G  GTR+G  GTR+G |
