# Supplementary material for: First appearance deceives many: disentangling the Hemidactylus triedrus species complex using an integrated approach
Source: PeerJ. 2018 Aug 2;6:e5341. doi: 10.7717/peerj.5341 (PMC6076986; doi:10.7717/peerj.5341)
Supplement: Supplemental Information 6 [file peerj-06-5341-s006.docx]

|  | NCBS AU703 | NCBS AU704 | NCBS AU705 | NCBS AU706 | NCBS AU707 | BNHS 1-18 | BNHS 849 | BNHS 683 | BNHS 683-1 | BNHS 1253 | BNHS 1668 | Min-Max | Mean | SD± |
| --- | --- | --- | --- | --- | --- | --- | --- | --- | --- | --- | --- | --- | --- | --- |
| Sex | ♂ | ♂ | ♂ | ♂ | ♂ | ♂ | ♂ | ♀ | ♀ | ♂ | ♂ |  |  |  |
| SVL | 72.1 | 68.9 | 65.9 | 63.4 | 74.4 | 74.3 | 68.2 | 60.6 | 58.8 | 58.7 | 76.7 | 58.7-76.7 | 67.45 | 7.3 |
| TRL | 30.2 | 31.3 | 28.2 | 26.3 | 29.6 | 30.2 | 31.3 | 30.4 | 26.8 | 25.8 | 34.1 | 25.8-34.1 | 29.47 | 3.0 |
| BW | 17.2 | 13.5 | 13.1 | 13.2 | 16.8 | 14.4 | 16.2 | 14.3 | 13.1 | 11.8 | 16.0 | 11.8-17.2 | 14.51 | 1.6 |
| CL | 10.6 | 11.0 | 9.4 | 8.6 | 8.8 | 10.0 | 10.5 | 9.0 | 9.2 | 7.7 | 9.8 | 7.7-11.0 | 9.51 | 0.9 |
| TL | - | 30* | 50* | 43.4* | 46.4* | - | 60.79* | - | 70.6 | - | 61.27* | - | - | - |
| TW | - | 7.6 | 6.5 | 8.2 | 9.3 | 8.1 | 8.2 | 7.8 | 7.7 | 7.4 | 7.4 | 7.4-9.3 | 7.82 | 0.8 |
| HL | 20.3 | 19.3 | 17.0 | 17.2 | 19.8 | 24.8 | 22.8 | 18.8 | 18.2 | 18.8 | 23.2 | 18.8-24.8 | 20.02 | 2.5 |
| HW | 16.3 | 15.7 | 14.6 | 14.1 | 17.8 | 15.0 | 16.4 | 14.1 | 13.8 | 12.9 | 15.4 | 12.9-17.8 | 15.10 | 1.7 |
| HH | 10.9 | 11.5 | 9.7 | 9.7 | 11.6 | 10.1 | 9.6 | 7.3 | 7.9 | 6.8 | 9.6 | 6.8-11.6 | 9.52 | 1.9 |
| FL | 10.3 | 9.6 | 6.8 | 6.7 | 9.3 | 9.4 | 10.2 | 8.9 | 8.9 | 7.7 | 10.3 | 7.7-10.3 | 8.92 | 1.4 |
| OD | 4.1 | 4.1 | 4.3 | 3.3 | 4.3 | 4.5 | 5.1 | 4.1 | 4.3 | 3.6 | 3.7 | 3.6-5.1 | 4.13 | 0.5 |
| NE | 7.2 | 6.2 | 5.7 | 5.9 | 6.4 | 6.4 | 6.6 | 5.4 | 5.5 | 5.5 | 5.2 | 5.5-7.2 | 6.00 | 0.7 |
| SE | 8.6 | 9.2 | 7.7 | 7.6 | 8.8 | 8.8 | 8.6 | 7.9 | 7.6 | 6.6 | 7.4 | 6.6-9.2 | 8.07 | 0.8 |
| EE | 7.0 | 6.7 | 5.5 | 6.0 | 7.2 | 7.2 | 6.9 | 5.7 | 5.6 | 5.5 | 5.6 | 5.5-7.2 | 6.26 | 0.9 |
| EL | 1.5 | 1.6 | 1.3 | 1.2 | 1.8 | 1.7 | 1.8 | 1.9 | 1.5 | 1.3 | 1.9 | 1.3-1.9 | 1.59 | 0.3 |
| IN | 2.1 | 2.8 | 2.5 | 2.5 | 2.7 | 2.3 | 2.0 | 2.0 | 2.1 | 1.9 | 1.9 | 1.9-2.8 | 2.25 | 0.3 |
| IO | 5.8 | 6.5 | 5.8 | 5.7 | 6.7 | 6.9 | 7.2 | 6.2 | 6.2 | 5.4 | 6.2 | 5.4-7.2 | 6.24 | 0.5 |
| Pores L | 7 | 8 | 7 | 8 | 9 | 9 | 8 | - | - | 7 | 8 | 7-9 | - | - |
| Pores R | 7 | 8 | 7 | 8 | 8 | 8 | 8 | - | - | 7 | 8 | 7-8 | - | - |
| Non-pored scales | 3 | 1 | 2 | 2 | 2 | 3 | 2 | - | - | 3 | 2 | 1-3 | - | - |

Table 1. Morphological and meristic data for specimens of *Hemidactylus triedrus*. ‘*’ indicates broken or regenerated tail.
