# Supplementary material for: First appearance deceives many: disentangling the Hemidactylus triedrus species complex using an integrated approach
Source: PeerJ. 2018 Aug 2;6:e5341. doi: 10.7717/peerj.5341 (PMC6076986; doi:10.7717/peerj.5341)
Supplement: Supplemental Information 7 [file peerj-06-5341-s007.docx]

| Specimens number | NCBS AU710 | NCBS AU711 | NCBS AU712 | NCBS AU713 | BNHS 1564 | BNHS 94 | BNHS 1478 | BNHS 96 | BNHS 93 | Min-Max | Mean | SD± |
| --- | --- | --- | --- | --- | --- | --- | --- | --- | --- | --- | --- | --- |
| Sex | ♂ | ♂ | ♂ | ♀ | ♂ | ♂ | ♀ | ♀ | ♀ | - | - | - |
| SVL | 78.0 | 68.0 | 56.0 | 63.0 | 67.6 | 68.7 | 71.1 | 72.5 | 61.2 | 56.0-78.3 | 66.6 | 7.3 |
| TRL | 30.3 | 21.0 | 25.0 | 33.0 | 27.0 | 31.3 | 29.3 | 35.4 | 24.4 | 21.0-35.4 | 28.4 | 4.0 |
| BW | 12.4 | 12.8 | 9.2 | 11.7 | 15.5 | 13.2 | 15.8 | 15.6 | 11.5 | 9.2-17.8 | 13.6 | 2.1 |
| CL | 11.0 | 9.9 | 7.8 | 10.7 | 9.3 | 10.3 | 10.5 | 10.4 | 9.5 | 6.6-12.7 | 9.9 | 1.5 |
| TL | 60* | 32* | 61.0 | 76.0 | 70.54* | - | - | 70.40* | - | - | - | - |
| TW | 7.8 | 6.6 | 5.0 | 5.4 | 7.5 | 9.0 | 8.2 | 9.0 | 7.2 | 5.0–9.5 | 7.1 | 1.4 |
| HL | 20.7 | 18.7 | 15.7 | 17.0 | 22.5 | 21.3 | 21.5 | 23.3 | 20.3 | 14.4–23.4 | 19.5 | 2.8 |
| HW | 17.2 | 14.3 | 11.4 | 13.9 | 17.0 | 15.0 | 14.1 | 15.7 | 12.4 | 11.4–17.3 | 14.4 | 1.8 |
| HH | 11.6 | 9.6 | 8.0 | 8.9 | 9.4 | 8.7 | 9.0 | 9.9 | 8.2 | 6.3–11.6 | 8.8 | 1.3 |
| FL | 10.5 | 9.4 | 6.8 | 10.0 | 8.3 | 9.8 | 9.6 | 9.8 | 8.7 | 6.6–12.9 | 9.4 | 1.5 |
| OD | 5.5 | 5.2 | 3.7 | 4.2 | 3.5 | 3.8 | 4.4 | 3.8 | 4.1 | 3.5–5.5 | 4.4 | 0.6 |
| NE | 6.3 | 5.2 | 5.3 | 5.4 | 7.0 | 6.3 | 6.2 | 7.1 | 5.3 | 4.7–8.0 | 6.1 | 0.9 |
| SE | 10.0 | 8.4 | 6.7 | 7.9 | 8.0 | 8.0 | 8.2 | 9.0 | 7.6 | 6.7–10.0 | 8.2 | 0.9 |
| EE | 7.2 | 5.8 | 4.9 | 4.9 | 6.7 | 6.1 | 6.4 | 6.6 | 5.4 | 4.9–7.2 | 6.0 | 0.7 |
| EL | 1.3 | 1.6 | 1.0 | 1.5 | 1.0 | 1.9 | 1.2 | 1.5 | 1.3 | 1.0–2.7 | 1.6 | 0.5 |
| IN | 3.2 | 2.4 | 1.5 | 1.8 | 1.9 | 2.3 | 2.0 | 2.0 | 2.2 | 1.5–3.2 | 2.2 | 0.4 |
| IO | 6.9 | 7.1 | 4.1 | 4.9 | 8.9 | 5.5 | 6.7 | 5.5 | 5.6 | 4.1–9.7 | 6.8 | 1.6 |
| Pores L | 13 | 12 | 13 | - | 11 | 11 | - | - | - | 11–15 | - | - |
| Pores R | 13 | 13 | 14 | - | 5+4 | 12 | - | - | - | 12–15 | - | - |
| gap btw pores | 1 | 1 | 1 | - | 3 | 1 | - | - | - | 1–3 | - | - |

Table 4. Morphological and meristic data for specimens of *Hemidactylus sahgali* **sp. nov.**  ‘*’ indicates broken or regenerated tail.
