# Supplementary material for: First appearance deceives many: disentangling the Hemidactylus triedrus species complex using an integrated approach
Source: PeerJ. 2018 Aug 2;6:e5341. doi: 10.7717/peerj.5341 (PMC6076986; doi:10.7717/peerj.5341)
Supplement: Supplemental Information 8 [file peerj-06-5341-s008.docx]

# Max likilhood partition

Species 1 (support = 0.929)

*Hemidactylus_depressus_1,Hemidactylus_depressus_2*

Species 2 (support = 1.000)

*Hemidactylus_prashadi_2*

Species 3 (support = 1.000)

*Hemidactylus_hunae*

Species 4 (support = 1.000)

*Hemidactylus_maculatus*

Species 5 (support = 0.744)

*Hemidactylus_triedrus_2,Hemidactylus_triedrus_1*

Species 6 (support = 0.987)

*Hemidactylus_sahgali_3,Hemidactylus_sahgali_2,Hemidactylus_sahgali_1*

Species 7 (support = 0.881)

*Hemidactylus_whitakeri_6*

Species 8 (support = 0.469)

*Hemidactylus_whitakeri_5,Hemidactylus_whitakeri_4*
