# Supplementary material for: First appearance deceives many: disentangling the Hemidactylus triedrus species complex using an integrated approach
Source: PeerJ. 2018 Aug 2;6:e5341. doi: 10.7717/peerj.5341 (PMC6076986; doi:10.7717/peerj.5341)
Supplement: Supplemental Information 11 [file peerj-06-5341-s011.docx]

| **Present work** | (Bauer et al., 2010) | (Bansal & Karanth, 2010) | (Chaitanya, Lajmi & Giri, 2018) |
| --- | --- | --- | --- |
| *H. sahgali* **sp. nov.** | *H. triedrus* | - | *H. triedrus* |
| *H. triedrus* | *H. lankae* | - | *H. triedrus/ H. lankae* |
| *H. whitakeri* **sp. nov.** | *H. subtriedrus* | *H. triedrus* | *H. subtriedrus* |

Table S8. Comparison of taxonomy of *H. triedrus* group

**References**

Bansal R., Karanth KP. 2010. Molecular Phylogenetics and Evolution Molecular phylogeny of *Hemidactylus* geckos (Squamata: Gekkonidae) of the Indian subcontinent reveals a unique Indian radiation and an Indian origin of Asian house geckos. *Molecular Phylogenetics and Evolution* 57:459–465. DOI: 10.1016/j.ympev.2010.06.008.

Bauer AM., Jackman TR., Greenbaum E., Giri VB., Silva A De. 2010. South Asia supports a major endemic radiation of *Hemidactylus* geckos. *Molecular Phylogenetics and Evolution* 57:343–352. DOI: 10.1016/j.ympev.2010.06.014.

Chaitanya R., Lajmi A., Giri VB. 2018. A new cryptic, rupicolous species of Hemidactylus Oken, 1817 (Squamata: Gekkonidae) from Meghamalai, Tamil Nadu, India. *Zootaxa* 4374:49–70. DOI: 10.11646/zootaxa.4374.1.3.
